# Regional biogeography versus intra-annual dynamics of the root and soil microbiome

**DOI:** 10.1186/s40793-023-00504-x

**Published:** 2023-06-07

**Authors:** Lukas P. Bell-Dereske, Gian Maria Niccolò Benucci, Pedro Beschoren da Costa, Gregory Bonito, Maren L. Friesen, Lisa K. Tiemann, Sarah E. Evans

**Affiliations:** 1 W.K. Kellogg Biological Station, Michigan State University, Hickory Corners, MI USA; 2 The Great Lakes Bioenergy Research Center, Michigan State University, East Lansing, MI USA; 3 Department of Plant, Soil and Microbial Sciences, Michigan State University, East Lansing, MI USA; 4 Laboratory of Entomology, Department of Plant Sciences, Wageningen University & Research, Wageningen, The Netherlands; 5 Department of Plant Pathology, Department of Crop and Soil Sciences, Washington State University, Pullman, WA USA; 6 Department of Integrative Biology, Michigan State University, East Lansing, MI USA; 7 Department of Microbiology & Molecular Genetics, Michigan State University, East Lansing, MI USA; 8 Laboratory of Environmental Microbiology, Institute of Microbiology, Czech Academy of Sciences, Vídeňská, Prague 4, 1083, 142 20 Czech Republic

**Keywords:** *Panicum virgatum*, Plant microbiome, Root bacteria, Root fungi, Soil bacteria, Soil fungi

## Abstract

**Background:**

Root and soil microbial communities constitute the below-ground plant microbiome, are drivers of nutrient cycling, and affect plant productivity. However, our understanding of their spatiotemporal patterns is confounded by exogenous factors that covary spatially, such as changes in host plant species, climate, and edaphic factors. These spatiotemporal patterns likely differ across microbiome domains (bacteria and fungi) and niches (root vs. soil).

**Results:**

To capture spatial patterns at a regional scale, we sampled the below-ground microbiome of switchgrass monocultures of five sites spanning > 3 degrees of latitude within the Great Lakes region. To capture temporal patterns, we sampled the below-ground microbiome across the growing season within a single site. We compared the strength of spatiotemporal factors to nitrogen addition determining the major drivers in our perennial cropping system. All microbial communities were most strongly structured by sampling site, though collection date also had strong effects; in contrast, nitrogen addition had little to no effect on communities. Though all microbial communities were found to have significant spatiotemporal patterns, sampling site and collection date better explained bacterial than fungal community structure, which appeared more defined by stochastic processes. Root communities, especially bacterial, were more temporally structured than soil communities which were more spatially structured, both across and within sampling sites. Finally, we characterized a core set of taxa in the switchgrass microbiome that persists across space and time. These core taxa represented < 6% of total species richness but > 27% of relative abundance, with potential nitrogen fixing bacteria and fungal mutualists dominating the root community and saprotrophs dominating the soil community.

**Conclusions:**

Our results highlight the dynamic variability of plant microbiome composition and assembly across space and time, even within a single variety of a plant species. Root and soil fungal community compositions appeared spatiotemporally paired, while root and soil bacterial communities showed a temporal lag in compositional similarity suggesting active recruitment of soil bacteria into the root niche throughout the growing season. A better understanding of the drivers of these differential responses to space and time may improve our ability to predict microbial community structure and function under novel conditions.

**Supplementary Information:**

The online version contains supplementary material available at 10.1186/s40793-023-00504-x.

## Introduction

Below-ground microbial communities are functionally important in ecosystems and respond strongly to plant communities. Roots, and their associated rhizospheres, are hot spots of activity with temporal changes in root exudate quality and quantity driving hot moments that can alter nutrient cycling [1]. Soils contain the majority of terrestrially stored carbon, and soil microbial communities are important drivers of carbon and nutrient dynamics below-ground [2]. Thus, a better understanding of the spatiotemporal patterns in below-ground microbial communities will likely improve our understanding of ecosystem productivity. However, previous studies of spatiotemporal patterns in plant microbiome niches (root and soil communities) have been confounded by plant community compositional changes. For example, at global and regional scales, soil pH and climate are frequently cited as drivers of the plant microbiome [3–6]. Soil pH and climate, along with other site-specific factors, also affect plant community composition and in turn plant community composition can affect soil biogeochemistry; for example, litter from Pinaceae decreasing soil pH [7], and legumes increasing soil nitrogen (N;[8]). These feedbacks can make it difficult to decouple the drivers of below-ground microbial communities. Furthermore, seasonal and annual shifts in microbial communities may differ between host plant species [9, 10]. Temporally, differential responses of microbial communities may be driven by host phenology and/or the interaction between host plants and site-specific conditions.

Bacterial and fungal communities of roots and soils play important roles in ecosystem productivity but, importantly, may respond differentially to spatiotemporal factors due to differences in their life history traits. Given their small size and single cell nature, bacteria tend to be much less dispersal limited than filamentous fungi, leading to differential spatiotemporal patterns [11]. These differences in dispersal limitation can lead to bacterial communities displaying more signs of environmental selection, such as differences in soil biogeochemistry across space [12] and less stochastic community assembly, compared to fungal communities [13, 14]. Differences in dispersal ability may also affect community responses to temporal factors with increased likelihood of stochastic patterns in succession of the fungal communities [15]. This may lead to mismatches in host versus microbial colonization of new locations [11] as has been demonstrated with ectomycorrhizal tree invasions [16]. However, it may be difficult to separate the effects of plant community composition from dispersal-dependent effects if these two factors co-vary. For example, although fungal communities tend to respond more strongly to plant community succession [17], the absence of nearby source fungal propagules may lead to reduced responsiveness to plant presence and identity, as compared to bacteria [13]. To better understand the drivers of spatiotemporal change in microbial communities, it is important to control plant host identity.

The widespread planting of perennial cropping systems offers an ideal opportunity to explore the spatiotemporal patterns in microbial communities while holding plant host species and genotype constant. The use of perennial cropping systems for food and biofuel production has been gaining attention recently as an alternative, or companion to, annual cropping systems [18]. Perennial cropping systems offer an opportunity to explore spatiotemporal turnover of below-ground microbial communities that have developed over multiple years while removing the confounding effects of plant species-site-time interactions. A better understanding of the spatiotemporal dynamics of the below-ground communities can also inform sustainable management and microbial assembly considerations that may be important to mitigating ongoing climate change [19]. Understanding and harnessing the below-ground microbial communities could also help off-set greenhouse gas production from agricultural systems, including reduced N_2_O production with reduced inorganic N inputs [20] and more sustainable production of cellulosic biofuel crops.

Application of N fertilizers may increase agricultural yields, but also may reduce the benefits of perennial crops, alter the microbial community, or alter plant-microbial interactions. Meta-analyses of the effects of N on microbial communities have found both positive and negative effects on microbial biomass and diversity. These variable results appear to be driven by the duration of the experiment, crop type, other nutrients included in the fertilizer, and the fertilizer application rate [21–23]. For example, N addition increased microbial diversity only when coupled with phosphorus and potassium, but decreased diversity when added alone [22]. Furthermore, microbial responses to N addition seemingly depend on treatment duration with reductions in microbial biomass in studies 5–10 years in length, but longer-term treatments led to increases in microbial biomass with peak increases found in studies of > 20 years in length [21]. However, the majority of studies of the effects of N addition on cropping systems have focused on annual crops. Therefore, the responses of perennial cropping systems are less well known.

After long-term N additions to a successional grassland at a site adjacent to the current study, N addition didn’t change microbial community diversity, but excess N addition, > 10.1 g N/m^2^, did affect community structure [24]. Previous research at our sites found that year to year variation in bacterial richness and microbial biomass outweighed the effects of N addition [25]. It has also been shown that excessive N in agricultural soils can increase the sensitivity of the microbial communities to seasonal dynamics and alter the interactions within microbial communities [26]. On the other hand, N addition may actually reduce the long-term variability of the microbial community [27] by creating long-term increases in plant productivity, and therefore increased plant carbon inputs to soils, and a stable resource base for microbes [21, 28]. Exploring an extensively distributed perennial cropping system under relatively long-term N fertilization (5 y) could improve our understanding of the effects of N application on microbial communities across spatiotemporal scales and help us resolve the apparent inconsistencies in the results described above.

Here we characterize the composition and spatiotemporal patterns in two niches (roots and soils) and domains (bacteria and fungi) in the microbiome of switchgrass monocultures, a perennial bioenergy crop species planted at five locations across Michigan and Wisconsin. Switchgrass is a primary candidate for next-generation cellulosic bioenergy [29], and a beneficial microbiome could contribute to its viability, but there have been few comprehensive microbiome characterizations across space and time [30]. To characterize temporal changes in microbial communities, we sampled a single site at 2-week (soil communities) and monthly (root communities) intervals. First, we hypothesized that N addition will have strong and consistent effects altering the structure of microbial communities across the five sites with the effects peaking post N application. Second, we hypothesize that the sampling site will strongly structure microbial communities through broadscale differences in climate and soil characteristics across this geographic region. Third, we hypothesize that the root microbial community structure would be more responsive than soil communities to collection date over the growing season due to the response of the root community to host phenology. Finally, to contribute to the fundamental knowledge of bioenergy crop microbiomes, we characterized a core switchgrass microbial community that is shared across sites, and a growing season.

## Methods

### Focal plant host

Switchgrass (*Panicum virgatum*, L.) is a flagship biofuel perennial species native to North America that has shown promise for sustainable production. One trait that makes switchgrass particularly attractive as a biofuel crop is that it can be productive in bioenergy lands, lands that are deemed not productive enough to grow conventional agricultural crops [31]. The below-ground microbial community of switchgrass may be a driver of this hardiness since it has been shown to have the potential to alleviate environmentally imposed stresses, including low N [32], as well as increasing water use efficiency and biomass of seedlings [33]. Yet, we are only beginning to understand the spatiotemporal distributions and biodiversity of the microbial communities that associate with switchgrass [30, 34].

### Study sites and treatments

We sampled the roots and bulk soils (hereafter, soils) of established switchgrass monocultures from the Marginal Land Experiment (MLE) of the Great Lakes Bioenergy Research Center (GLBRC: www.glbrc.org). For a full description of its establishment and ongoing treatments see https://data.sustainability.glbrc.org/pages/1.html#marginal. For a description of soil health, and other site level characteristics, see Li et al. [25] and Table S1 & S2. We sampled switchgrass monocultures (G5 treatment), which were established in 2013 by sowing 0.78 g/m^2^ of Cave-in-Rock variety seeds with four plot replicates at each site. Each plot was subdivided into N addition and control (no N addition) split-plots (hereafter, subplots). Nitrogen addition subplots received 5.6 g N/m^2^ at the beginning of each growing season (late May) starting in 2015 with Michigan sites receiving SUPERU (Koch Agronomic Services, Wichita, KS, USA) and Wisconsin sites receiving ESN (Nutrien, Saskatoon, Canada) slow-release urea fertilizers. Plots were managed based on recommended agronomic practices with the use of herbicides and pelletized lime to maintain switchgrass monocultures and soil pH, respectively (see https://data.sustainability.glbrc.org/datatables/204).

To characterize the switchgrass microbiome at a regional scale, we sampled the five MLE sites of Michigan and Wisconsin in July 2018 (three Michigan and two Wisconsin sites). These sites represent ~ 3.3 degrees of latitude and ~ 4.3 degrees of longitude. Plots were 19.5 m x 19.5 m at Lake City and Escanaba and 19.5 m x 12.2 m at Lux Arbor, Hancock and Rhinelander. Block 1 at Hancock was decommissioned in 2015 leaving three replicates at the site (Table S3).

Root and soil samples were temporally paired by shared collection days and spatially paired by sampling points. To capture the switchgrass microbiome across the growing season (i.e., temporal patterns), we collected roots and soils from Lux Arbor, the southernmost site in Michigan, during the 2018 growing season. Soils were collected every two weeks between March 19th and November 5th. Roots were collected every four weeks from May 29th to October 3rd, on days coinciding with soil collections (Table S4).

### Plant microbiome sampling

Soil sampling was conducted following methods described in [35]. Briefly, cores (2.5 cm wide, 10 cm deep) were collected from three predetermined random sampling points within each N addition and control subplot. Roots were collected from 2 – 3 mini-cores (1.27 cm wide, 10 cm deep) taken near each soil core sampling point. This resulted in six root and six soil samples per plot per sampling effort (total of 24 samples per niche per sampling effort). Coordinates for each core were converted to universal transverse Mercator coordinates based on the estimated distance to the southeast corner of each plot using the ‘rgdal’ and ’sp’ packages in R [36–39]. These coordinates were used for all pairwise measures of spatial distance. Soil samples were transported and stored at 4 °C then roots and large particulate matter were removed through sieving with a 4-mm mesh sieve within three days of collection. Finally, soils were stored at -80 °C at the Kellogg Biological Station (Hickory Corners, MI, USA). Roots were transported at 4 °C and processed at Michigan State University (MSU; East Lansing, MI, USA). Roots were separated from soil using bleach-sterilized tweezers and sieves, washed in a 0.5% Tween20 solution to remove soil debris, rinsed three times with sterile deionized water, flash-frozen in liquid N, and then lyophilized.

### Microbial community characterization

Before DNA was extracted from roots with the Mag-Bind plant kit (Omega bio-Tek Inc., Norcross, GA, USA), lyophilized roots were powderized using sterile tungsten beads on a TissueLyser II robot (QIAGEN, Hilden, Germany). DNA from soils was extracted in 96-well plates using the KingFisher Flex Purification System (Thermo Fisher Scientific, Waltham, MA, USA) with the MagAttract PowerSoil DNA KF kit (QIAGEN). For bacteria, the V4 hypervariable region of 16 S rRNA gene was amplified using 515 F/806R primers [40]. This primer set amplifies both bacteria and archaea, but we refer to the community as bacteria for simplicity. Illumina compatible libraries were prepared for soil bacterial communities using primers containing both the target sequences and the dual indexed Illumina compatible adapters [41] by the MSU Research Technology Support Facility (RTSF) Genomics core. Fungal ITS1 rDNA was amplified with the primer pair ITS1F/ITS2 [42]. Libraries for root bacterial communities and both fungal communities were multiplexed following a three-step PCR sequence as described in [43]. The completed libraries were normalized with Invitrogen SequalPrep DNA Normalization plates (Thermo Fisher Scientific), pooled and cleaned up with AmpureXP magnetic beads (Beckman Coulter, Brea, CA, USA). Libraries were then paired-end sequenced by MSU RTSF Genomics core on a MiSeq platform (Illumina Inc., San Diego, CA, USA) using the v2 kit for soil bacterial libraries and the v3 kit for root bacterial and fungal libraries.

Diversity and richness of soil bacteria and fungi from MLE sites were partially published in [25]. However, sequences were reprocessed and re-clustered here (see below) to allow for comparisons between roots and soils. Additionally, Lux Arbor samples used in Li et al. were collected on July 9th [25] while we used samples from July 30th for our regional comparisons to match the timing of root sampling. Soil bacterial libraries were demultiplexed by MSU RTSF using Illumina bcl2fastq while root bacterial and fungal libraries were demultiplexed in QIIME 2 using “demux emp-paired” [44]. Post paired-end sequence merging, root and soil reads were pooled for bacteria and fungi separately. Pooled libraries were separately quality filtered, and clustered into operational taxonomic units (OTUs) using the USEARCH pipeline (http://drive5.com/usearch/; [45, 46]). Primers and adapters were removed, then bacterial sequences were filtered and trimmed to 250 bp while fungal sequences < 100 bp were removed but not trimmed. Sequences were then quality filtered (max EE < 1), clustered into OTUs at 97% sequence similarity, and taxonomically classified using SINTAX [46, 47]. Bacterial OTUs were classified against SILVAv123 rRNA database [48] and all OTUs classified to chloroplast and mitochondria were removed. Fungal OTUs were classified against UNITE v8.2 (04.02.2020) eukaryote database [49]. OTUs classified as non-fungal were removed, as were Malasseziomycetes since these fungi are well known to be human-associated [50]. Fungal OTUs were then reclassified with CONSTAX2 against UNITE v8.2 (04.02.2020) eukaryote database to increase the depth of taxonomic classifications [51]. Both community matrices were rarefied to 10,000 reads. A total of 7 bacterial and 18 fungal samples were removed from analyses due to poor quality or quantity of sequences (e.g., less than 10,000 reads) or because they were extreme outliers in NMDS space (one bacterial sample, Table S3 & S4). For post-processed and rarefied community richness and read abundances, see Table S5.

### Statistical analyses

All statistical analyses, with the exception of PERMANOVA, were conducted in R version 4.2.1 [39]. To characterize the effects of spatiotemporal factors on microbial community composition we constructed PERMANOVA models, based on Bray-Curtis (BC) distance, for combinations of domain (bacteria or fungi) and niche (roots or soils) in Primer v6 [52]. Models for the MLE communities tested the effects of site, N addition, and their interactions, with plot nested within site × N addition as a random factor, on community composition. Models for Lux Arbor tested the effects of collection date as a factor, N addition, and their interactions, with plot nested within date × N addition as a random factor, on community composition. PERMANOVA models used type 3 sums of squares and 9999 permutations to determine p-values. Effects sizes were calculated using the coefficient of determination (R^2^) and omega-squared (ω^2^) according to methods in [53]. Concordant variations in β-diversity between two communities can indicate co-occurrence or similar responses of both communities to the same environmental factors. To test if variation in community structure were concordant between bacteria and fungi, we used Procrustes analysis [54, 55], combined with permutation tests [56], using the *protest* function in ‘vegan’ with 9999 permutations [57]. Tests of the effects of spatiotemporal factors on richness (# of OTUs), diversity (inverse Simpson), and residuals of concordance between communities (bacterial and fungal communities) used the same model structures described above, with plot as a random factor, using mixed effects models. To test the similarity ($$ 1-BC dist$$) between root and soil communities collected either 2-weeks prior to root collection or on the same collection date as roots, we constructed mixed effects models with independent factors of collection date, N addition, soil comparison date (2-weeks or same day), and their interactions, with root sample nested within plot as a random factor. Importantly, though root and soil samples were spatially paired for same day comparisons, the comparisons between roots and soils collected 2-weeks prior was the mean similarity between root samples and soil samples collected from the same subplot. All mixed effects models were constructed with *lmer* in ‘lme4’ [58] with type 3 sums of squares using *anova* in ‘lmertest’ [59]. The normality and heteroscedasticity of model residuals were tested using the Shapiro–Wilk test and *simulateResiduals* in ‘DHARM’ [60] with data transformed when necessary. Pairwise statistical significance was calculated using ‘emmeans’ with Tukey honest significant difference [61].

Further testing the spatiotemporal dynamics of the microbial communities, we constructed generalized dissimilarity models (GDMs) testing the relationship between the pairwise distance in space and time to the BC pairwise distance between community compositions for the combinations of domain (bacteria or fungi) and niche (roots or soils) with samples from Lux Arbor. Models were constructed using the ‘gdm’ package [62, 63] with 95% confidence intervals surrounding the loess lines calculated using bootstrapping with 30% of the samples withheld in each of the 100 permutations. To explore potential drivers of the spatiotemporal dynamics, we constructed GDMs with soil nutrients (NO_3_, NH_4_, organic C and N, pH, K, Ca, P), plant traits (SLA, shoot and root biomass, subplot yield), and meteorological factors (MET: preceding seven-day rain accumulation, soil moisture and temperature) as factors for MLE sites (Table S1) and Lux Arbor growing season (Table S2). Outlier values were identified and removed based on field and lab notes combined with the removal of values greater than 3× the inter-quantile of data from 2016 to 2018 using *is_extreme* in ‘rstatix’ [64]. Missing values for potential drivers (Table S1 & S2) were imputed using a random forest imputation algorithm in the ‘missForest’ package [65]. We used the *gdm.varImp* to select factors for the final model based on backward selection of importance values with 100 permutations. Partitioning of the deviance explained was conducted using *gdm.partition.deviance* with MET, soil nutrient, and plant trait factors grouped [62, 63]. In order to fit the models, and allow for comparisons between niches, analyses of the soil communities of Lux Arbor were restricted to dates where roots were sampled (monthly sampling).

To characterize the core communities of switchgrass, we used methods detailed in [66], which combines occupancy-abundance and threshold effects of species removal on pairwise BC distance to identify the core community. Core community selection was conducted across sites and collection dates for the MLE and Lux Arbor communities, respectively. We chose a single threshold of 5% change in BC distance across bacteria and fungi in roots and soils. This single threshold enabled standardized comparisons across these groups and reduced the complexity of methodological biases across comparisons. We used code adapted from [67] to optimize the BC threshold based on the fit of neutral models (Fig. S1 - S4; [68]) and the effects of threshold on the richness and relative abundance of taxa included in the core community (Fig. S5 - S8). We examined the differences in the abundance of core taxa classified to known families between roots and soils using *heat_tree* in ‘metacoder’ [69]. Differential abundances were calculated using log2 ratio of median proportions with pairwise significance calculated using Wilcoxon rank-sum. Core taxa were then classified to guilds with FunGuild classifications of “Probable” and “Highly Probable” [70]. Taxa with multiple guild classifications were grouped into symbiotrophs (putative mutualists/commensalists; e.g., arbuscular mycorrhizal (AM) fungi), pathogens (putative antagonists; e.g., plant pathogens), or saprotrophs. These classifications were similar to FunGuild trophic groups but also highlighted instances of multiple guild classifications within a given group (e.g., “Multiple Saprotroph”; Table S6).

## Results

### Effect of N addition regionally and across the growing season

**Fig. 1:**
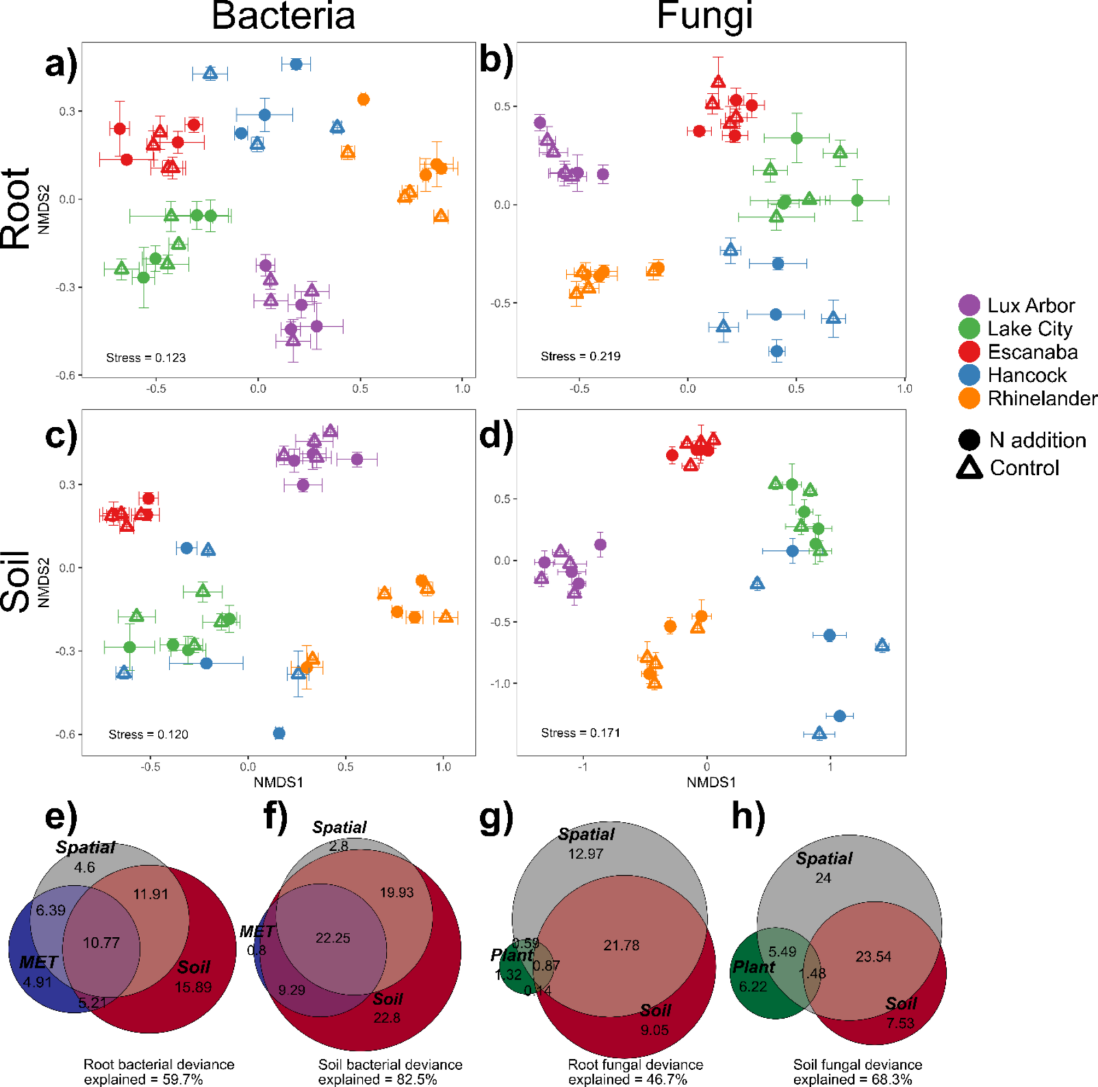
Microbiome compositions of Marginal Land Experiment (MLE) sites: NMDS plots of (**a**) root bacterial, (**b**) root fungal, (**c**) soil bacterial, and (**d**) soil fungal communities of switchgrass monocultures from MLE sites located in nitrogen (N) addition (filled circles) and control (open triangles) subplots; each point is averaged within subplot. Error bars are ± SE. Euler diagrams represent the partitioning of the deviance from generalized dissimilarity models of (**e**) root bacterial, (**f**) soil bacterial, (**g**) root fungal, and (**h**) soil fungal communities into spatial (pairwise distance between samples), soil (pH, total organic N (TON), K, and Ca), meteorological (MET: preceding seven rain day accumulation), and plant (root biomass and subplot yield)

Overall, N addition had little to no effect on the structure of the switchgrass below-ground microbiome. We found a significant effect of N addition on root bacterial composition across sites (Fig. 1a), but N only explained 1.7% of the variance and there were no significant effects of N on the other communities (*p* > 0.25; Table 1). Nitrogen addition also reduced the concordance between root bacterial and fungal communities of Rhinelander (Fig. S9c) but had no effect on the other sites or soil community concordances (Table S7; Fig. S9). Finally, effects of N addition on diversity were context dependent, N addition reduced overall root bacterial richness and diversity (Fig. S10ac), but increased root fungal diversity in Escanaba (Table S8; Fig. S10d).

**Table 1:** PERMANOVAs of switchgrass microbiome composition from the Marginal Land Experiment sites

		Root Bacteria				Root Fungi			
	df	Pseudo-F	R^2^	ω^2^	P-value	Pseudo-F	R^2^	ω^2^	P-value
Site	4	**11.53**	**0.373**	**0.354**	**< 0.001**	**7.41**	**0.292**	**0.271**	**< 0.001**
N add	1	**2.10**	**0.017**	**0.017**	**0.001**	1.06	0.010	0.010	0.359
Site*N add	4	1.08	0.035	0.008	0.250	0.72	0.028	0.001	0.993
Plot(Site*N add)	28	**1.75**			**< 0.001**	**1.86**			**< 0.001**
		Soil Bacteria				Soil Fungi			
	df	Pseudo-F	R^2^	ω^2^	P-value	Pseudo-F	R^2^	ω^2^	P-value
Site	4	**12.79**	**0.460**	**0.443**	**< 0.001**	**9.80**	**0.396**	**0.377**	**< 0.001**
N add	1	0.57	0.005	0.005	0.965	0.95	0.010	0.010	0.554
Site*N add	4	0.50	0.018	< 0.001	1.000	0.61	0.025	< 0.001	1.000
Plot(Site*N add)	28	**2.58**			**< 0.001**	**2.65**			**< 0.001**

Results from models testing the effects of site and nitrogen (N) addition on the composition of bacterial and fungal communities from roots and soils of switchgrass monocultures from the Marginal Land Experiment sites. Bolded texts highlight significant factors

**Fig. 2:**
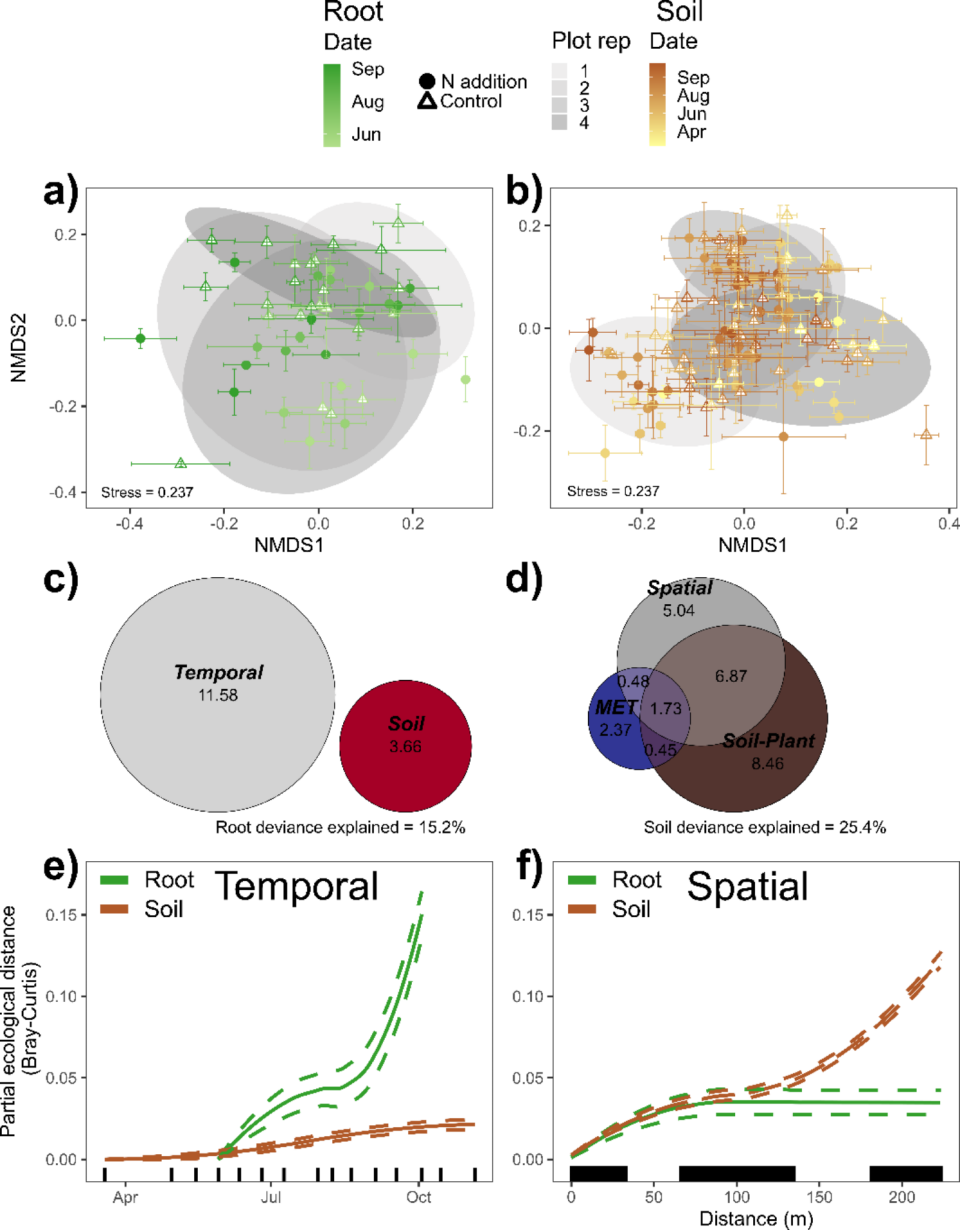
Bacterial community compositions across the growing season of Lux Arbor: NMDS plots of the (**a**) root and (**b**) soil communities of switchgrass. Symbols represent the mean composition in nitrogen (N) addition (filled circles) and control (open triangles) subplots with error bars representing SE. Fill color ramp represents collection dates with lighter colors representing earlier dates and darker colors later dates. Gray ellipses represent plot level composition at 95% confidence. Euler diagrams of (**c**) root bacterial and (**d**) soil bacterial communities represent the partitioning of the deviance from generalized dissimilarity models (GDMs) into spatial (pairwise distance between samples), temporal (collection date), soil (soil Ca), meteorological (MET: soil core gravimetric soil moisture), and soil-plant (subplot yield, soil Ca). Loess graphs from GDMs of (**e**) temporal and (**f**) spatial patterns in community change of root bacteria (green lines) and soil bacteria (brown lines). Dashed lines represent 95% confidence. Vertical lines along the x-axes represent collection dates and pairwise spatial distances

The effect of N addition on switchgrass growing season microbiome composition was significant but small, explaining 1.3 − 3.1% of the community variance, and did not interact with collection date (Table 2; Fig. 2ab & 3ab). Nitrogen addition reduced the concordance between bacterial and fungal community composition in both niches (Fig. S11) with N driven disruption the strongest for root bacterial and fungal communities at the end of the growing season (Table S9; Fig. S11ac). Similar to the regional results, the growing season effects of N addition on diversity were context dependent. Nitrogen addition increased soil bacterial richness and diversity (Fig. S12ac) but decreased root fungal diversity (Table S10; Fig. S12d).

**Table 2:** PERMANOVAs of switchgrass microbiome composition from across the growing season at Lux Arbor

		Root Bacteria				Root Fungi			
	df	Pseudo-F	R^2^	ω^2^	P-value	Pseudo-F	R^2^	ω^2^	P-value
Collection Date	5	**2.62**	**0.112**	**0.085**	**< 0.001**	**1.39**	**0.066**	**0.038**	**0.020**
N add	1	**2.54**	**0.022**	**0.022**	**< 0.001**	**2.61**	**0.025**	**0.024**	**0.003**
Date*N add	5	0.87	0.037	0.009	0.835	0.77	0.036	0.007	0.935
Plot(Date*N add)	6	**1.54**			**< 0.001**	**1.64**			**< 0.001**
		Soil Bacteria				Soil Fungi			
	df	Pseudo-F	R^2^	ω^2^	P-value	Pseudo-F	R^2^	ω^2^	P-value
Collection Date	14	**2.06**	**0.111**	**0.077**	**< 0.001**	1.11	0.073	0.037	0.179
N add	1	**3.33**	**0.013**	**0.013**	**< 0.001**	**6.57**	**0.031**	**0.031**	**< 0.001**
Date*N add	14	0.60	0.033	< 0.001	1.000	0.48	0.032	< 0.001	1.000
Plot(Date*N add)	6	**1.83**			**< 0.001**	**2.44**			**< 0.001**

Results from models testing the effects of collection date and nitrogen (N) addition on the composition of bacterial and fungal communities from roots and soils of switchgrass monocultures across the growing season at Lux Arbor. Bolded texts highlight significant factors

### Regional patterns in microbial composition

Site was the strongest determinant of microbial community structure, explaining > 27% of the variance in communities and niches (Table 1). According to deviance partitioning of the GDMs, soil variables, primarily soil pH, seemed to be the strongest drivers of the differences between sites (Fig. 1e-h & S13). In general, root communities were less structured by site than soil communities while bacterial communities were more structured by site than fungal communities (Table 1). Abiotic factors explained most of the model deviance in bacterial communities (Fig. 1ef), while pairwise distance between sites explained most of the model deviance in the fungal communities (Fig. 1gh), suggesting that stochastic spatial dynamics dominate fungal communities more than bacterial communities. Plant traits were important predictors of the fungal community composition (Fig. 1gh), specifically root biomass for root communities and subplot yield for soil communities (Fig. S13bd). However, no plant traits remained in the final bacterial models (Fig. 1ef). Additionally, no meteorological factors remained in the final fungal models (Fig. 1gh). The concordance between bacterial and fungal communities was affected by sampling site, with root communities of Rhinelander having the highest concordance (Fig. S9ac) and the soil communities of Hancock having significantly lower concordance compared to the other sites (Table S7; Fig. S9bd). Site also had a significant effect on beta dispersion in all communities (Table S11; Fig. S14). Finally, site had a strong effect on richness and diversity across both niches and domains (Table S8). Lux Arbor tended to support the highest richness across all communities (Fig. S10ab) and higher bacterial diversity while Rhinelander tended to have the lowest richness and diversity (Fig. S10).

### Changes in microbial communities across a growing season

**Fig. 3:**
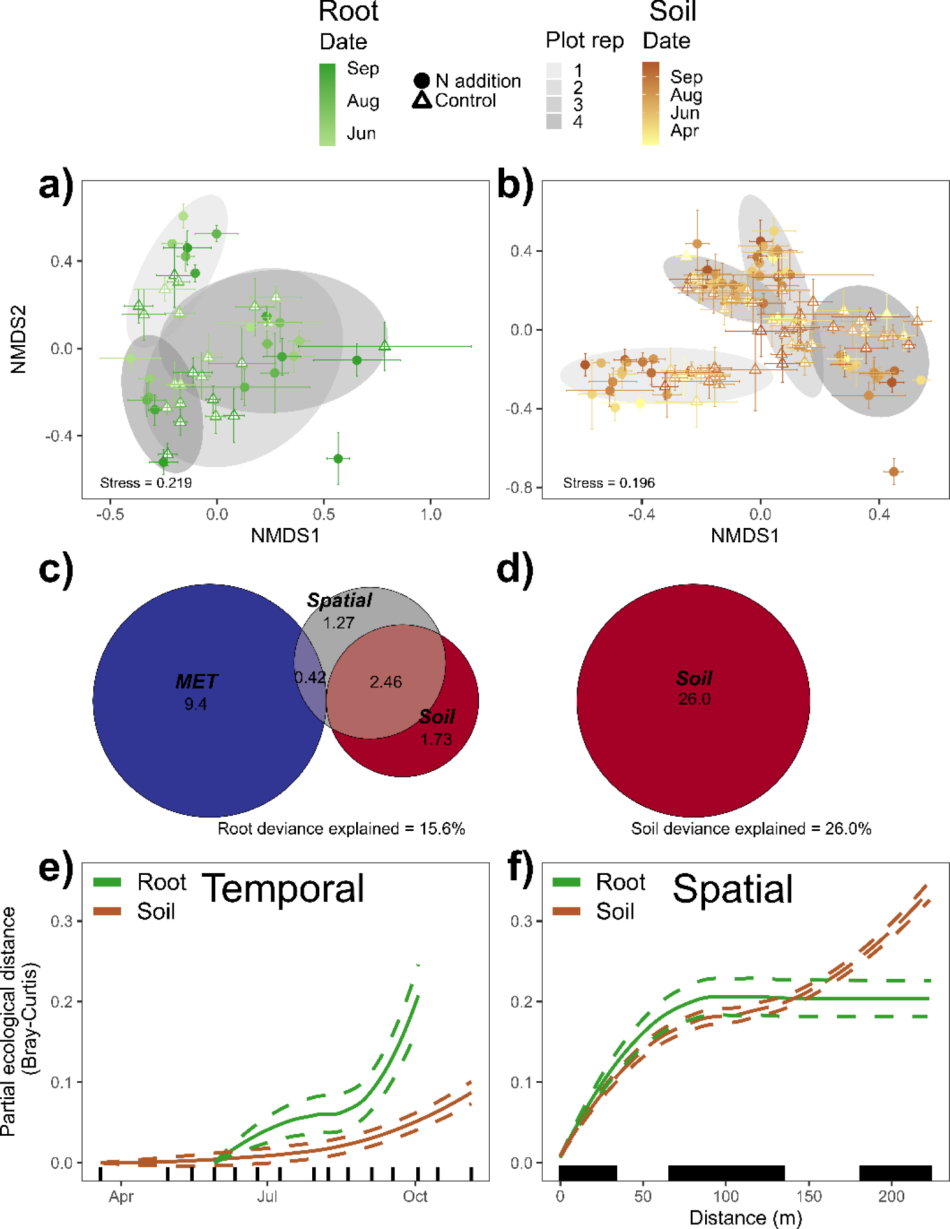
Fungal community compositions across the growing season of Lux Arbor: NMDS plots of the (**a**) root and (**b**) soil communities of switchgrass monocultures. Symbols represent the mean composition in nitrogen (N) addition (filled circles) and control (open triangles) subplots with error bars representing SE. Fill color ramp represents collection dates with lighter colors representing earlier dates and darker colors representing later dates. Gray ellipses represent plot level composition at 95% confidence. Euler diagrams of (**c**) root fungal and (**d**) soil fungal communities represent the partitioning of the deviance from generalized dissimilarity models (GDMs) into spatial (pairwise distance between samples), soil (soil pH, P, K, and Ca), and meteorological (MET: soil core gravimetric soil moisture and 24-hour average of soil temperature). Loess graphs from GDMs of (**e**) temporal and (**f**) spatial patterns in community change of root fungi (green lines) and soil fungi (brown lines). Dashed lines represent 95% confidence. Vertical lines along the x-axes represent collection dates and pairwise spatial distances

Across the Lux Arbor growing season, the majority of variance in community composition was explained by collection date (3.8 − 8.5%; *p* < 0.02; Fig. 2ab & 3a) with the exception of soil fungi which did not significantly vary across dates (*p* > 0.15; Table 2; Fig. 3b). Collection date was a significant predictor of soil fungal composition in the GDM but only explained 0.8% of deviance (Table 3) and did not remain in the final backward selected GDM (Fig. 3d & S15d). Geographical distance within Lux Arbor was a better predictor of soil bacterial and fungal community change than collection date (Table 3; Fig. 2ef & 3ef), while collection date was a better predictor of the community change for root bacteria (Fig. 2e). Supporting this result, collection date remained in the final root bacterial GDM explaining 11.6% of the community deviance (Fig. 2c). Importantly, the only plant trait that remained a significant predictor in the final GDMs of the switchgrass growing season microbiome was subplot yield in the soil bacterial models (Fig. S15c). Soil and MET factors, such as soil Ca and moisture, were more frequent predictors of the switchgrass microbiome (Fig. 2cd & 3 cd).

**Table 3:** Generalized dissimilarity models of switchgrass microbiomes from across the growing season at Lux Arbor

	Root Bacteria		Soil Bacteria		Root Fungi		Soil Fungi	
% Total deviance explained	12.90		15.54		7.21		15.06	
	% Partial deviance explained	P-value	% Partial deviance explained	P-value	% Partial deviance explained	P-value	% Partial deviance explained	P-value
Spatial	1.31	< 0.01	15.08	< 0.01	4.09	< 0.01	14.22	< 0.01
Temporal	11.46	< 0.01	0.43	0.11	3.00	< 0.01	0.79	0.03

Model fits of spatiotemporal change in the bacterial and fungal communities of roots and soils of switchgrass monocultures across the growing season at Lux Arbor

**Fig. 4:**
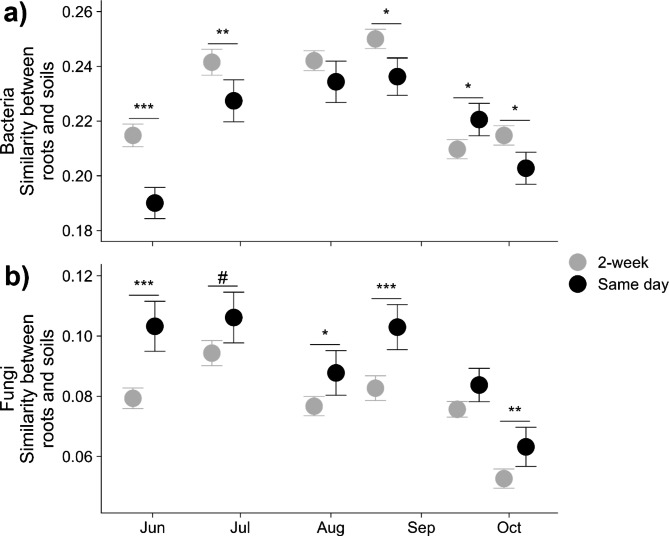
Similarity ($$ 1-Bray Curtis dist$$) between root and soil microbiomes across the growing season of Lux Arbor: a) bacterial and b) fungal root communities and soil communities collected either the same day as roots (same day) or soil communities collected two weeks prior to the root sampling (2-week). “#”, “*”, ”**”, and ”***” represents *p* < 0.10, *p* < 0.05, *p* < 0.01, and *p* < 0.001 Tukey HSD adjusted significance. Nitrogen addition did not have a significant effect on similarity and, for this reason, is not shown

The similarity in microbiome composition within and between niches was temporally dependent. Root bacterial communities were, in general, more similar to soils collected 2-weeks prior to root collection than same day collections (Fig. 4a) while root fungal communities were more similar to soils collected on the same day (Table S12; Fig. 4b). However, root bacterial communities collected on Sept. 17 were more similar to soils collected the same day compared to earlier soils (Fig. 4a). This corresponded with a decline in overall similarity between root and soil communities (Fig. 4) and an increase in community beta dispersion (Table S13; Fig. S16) toward the end of the growing season. The collection date had a significant effect on the concordance between root bacterial and fungal communities but no effect on soil communities (Table S9; Fig. S11).

### Characterization of a switchgrass core microbiome community

**Fig. 5:**
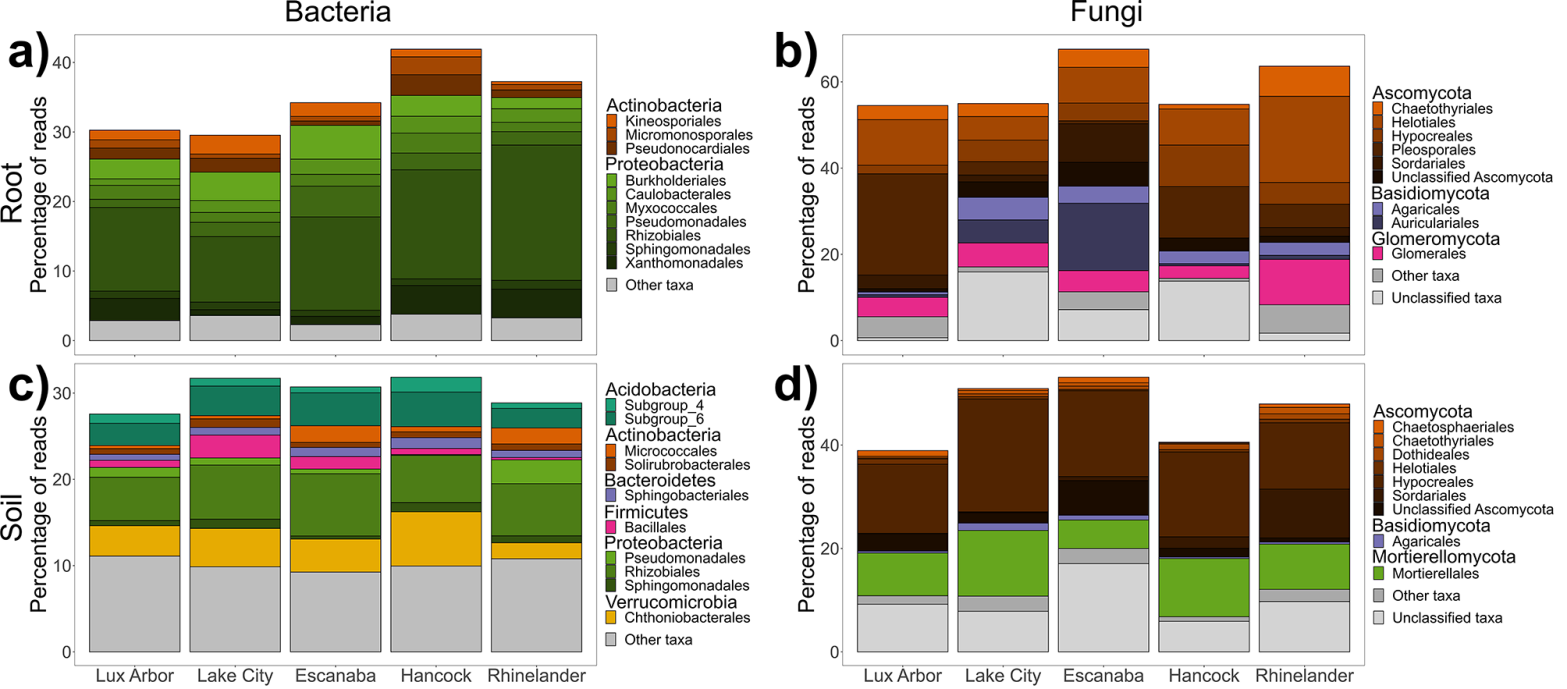
Stacked bar plots of the top ten most abundant orders across sites within core microbiomes (**a**) root bacterial, (**b**) root fungal, (**c**) soil bacterial, and (**d**) soil fungal communities of switchgrass monocultures at sites of the Marginal Land Experiment. Orders are grouped by phyla

**Fig. 6:**
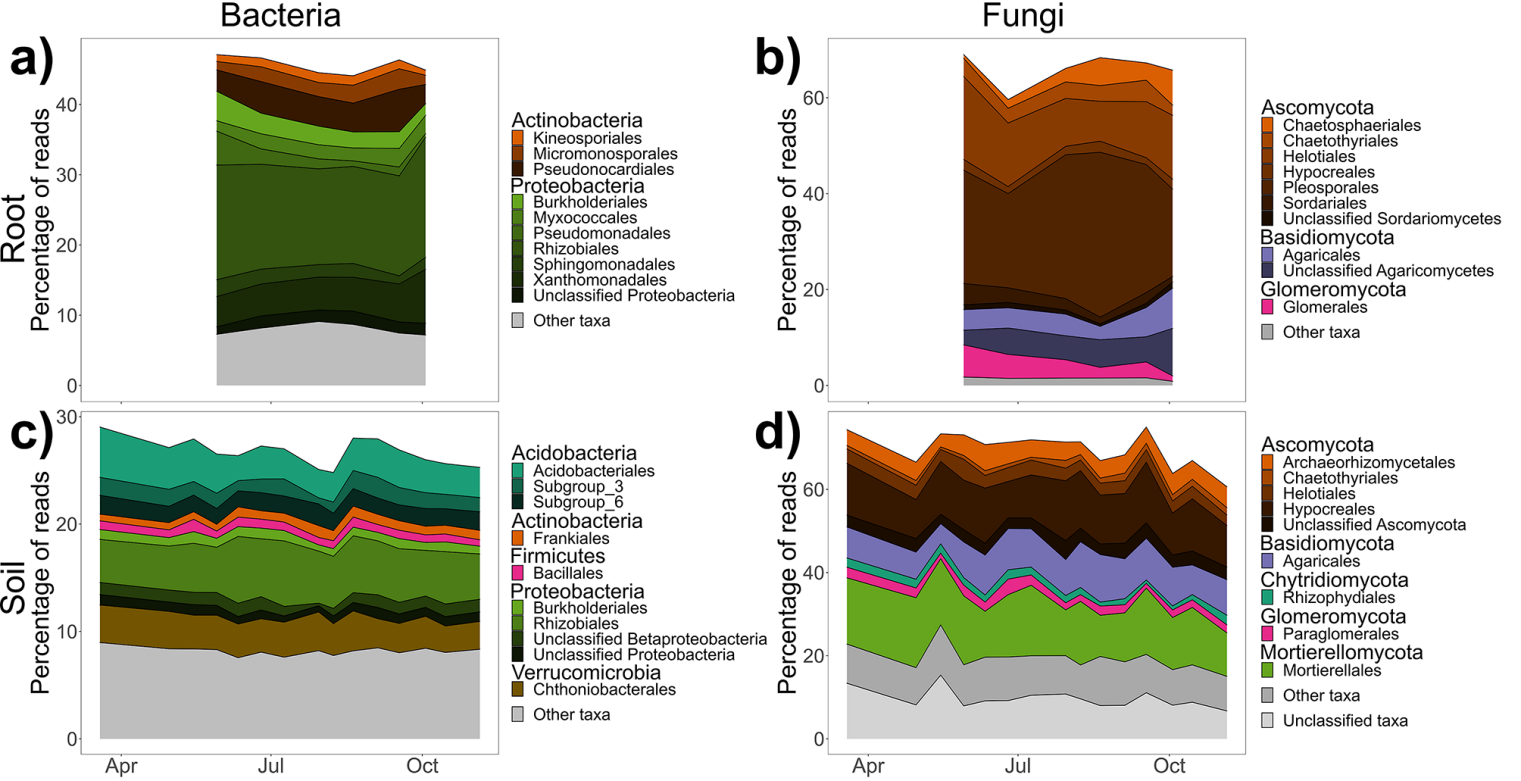
Stacked bar plots of the top ten most abundant orders across the growing season within core microbiomes (**a**) root bacterial, (**b**) root fungal, (**c**) soil bacterial, and (**d**) soil fungal communities of switchgrass monocultures from Lux Arbor across one growing season. Orders are grouped by phyla

We characterized a switchgrass core microbiome, taxa that persist across space and time, which future work may functionally characterize and optimize to benefit bioenergy crops. These core taxa made up greater than 34% and 27% of reads in the root and soil communities, respectively (Table S14). The core root bacterial community hosted less phylogenetic diversity and 100 fewer OTUs than the core soil bacterial community across the MLE sites (Fig. S17ac), but only 8 fewer OTUs in the Lux Arbor growing season communities (Table S14; Fig. S18ac). Rhizobiales were dominant members of the core bacterial communities across sites and the growing season (Supporting Information Core Community Supplementary Material 2) (Fig. 5ac & 6ac) and were a larger portion of the core root microbiome than core soil (Fig. S17ac & S18ac). At Lux Arbor, the relative abundance of Burkholderiales and Pseudomonadales was greatest at the start of the growing season while Xanthomonadales reached the highest relative abundance towards the end of the growing season (Fig. 6a). A *Bradyrhizobium* sp. (OTU1) dominated communities, accounting for ~ 10.3% of rarefied reads in the root communities while making up ~ 1.5 − 2.3% of rarefied reads in the soil communities across sites and the growing season (Supporting Information Core Community Supplementary Material 2).

Within the core fungal community, roots hosted twice the richness of OTUs compared to the soil communities across the MLE sites (Fig. S17bd), but roots hosted less than 50% of the richness found in soils across the growing season (Table S14; Fig. S18bd). Glomeraceae was the most abundant and rich classified family in the core root community (Fig. S17b & S18b). Hypocreales and Mortierellales dominated the core soil communities, but the dominant orders in the core root community varied between MLE sites (Fig. 5bd). Pleosporales, Auriculariales, and Helotiales dominated the Lux Arbor, Escanaba, and Rhinelander core root communities, respectively (Fig. 5b). The dominance of Pleosporales in the core root community of Lux Arbor was consistent across the growing season with the order reaching its highest abundance in August (Fig. 6b). Two Nectriaceae spp. were dominant in the core switchgrass microbiome (OTU1 and OTU142) across the MLE sites (Supporting Information Core Community Supplementary Material 2). The majority of fungal species could not be unambiguously classified to a primary guild in FunGuild (unknown guilds: 64 OTUs (43.0% of OTUs) and 95 OTUs (51.1% of OTUs) in regional and growing season core communities, respectively). The most dominant guilds in the core root microbiome were arbuscular mycorrhizal (AM) fungi, endophytes, and unidentified saprotrophs (Fig. S19a & S20a). The core root community of Escanaba was dominated by saprotrophs while the core root community of Lux Arbor was dominated by AM fungi and endophytes (Fig. S19a). The dominance of AM fungi in the Lux Arbor core root community declined towards the end of the growing season. Consequently, endophytes and taxa classified as multiple guilds spanning pathogens and saprotrophs were dominant by the end of the growing season (Fig. S20a). The core soil fungal community was dominated by taxa classified as saprotrophs and multiple guilds spanning saprotrophs and symbiotrophs (Fig. S19b & S20b).

## Discussion

Our study is among the first to simultaneously characterize the effects of N addition and spatiotemporal factors on the below-ground microbiome, across domains and niches, while controlling for plant species. Our results show that spatiotemporal factors explain more of the structure of the plant microbiome than N additions, which had surprisingly little effect on both soil and root microbial communities. Perhaps this was because of the relatively low N application rate in our system compared to other agricultural systems. Both bacteria and fungi, no matter the niche (root vs. soil), were strongly structured by the sampling site, highlighting the importance of soil and climate history on plant microbiome structure. The spatial location also influenced microbiomes within a site (0.01–250 m), especially for soil fungi. Importantly, while bacteria were more structured by sampling sites, and despite our extensive evaluation of 13 spatiotemporally structured environmental factors, fungal community composition had less spatial structure that could be explained by environmental factors indicating that stochastic factors, such as dispersal limitation, are likely dominant drivers. Even though we found spatiotemporal variation, we identified a functionally important core microbial community that was relatively stable across sites and growing season, with the exception of the core root fungal community which was more spatiotemporally variable.

### Greatest variation in all microbiomes seen at large spatial scales

Overall, we found that the sampling site explained the greatest portion of microbiome structure across the microbial niches (roots and soil) and domains (bacteria and fungi; Table 1). Previous research has found strong spatial patterns in below-ground microbial community compositions at similar regional [3, 71] and global scales [5, 6, 72]. Interestingly, a survey of switchgrass monocultures in North Carolina over a similar geographic extent (about 460 km) found that both root and soil fungal communities were most strongly structured by spatial processes occurring at less than half of a kilometer scale, with both roots and soils showing similar scales of structure [34]. While we did find a significant effect at < 250 m scale at Lux Arbor, communities were much more structured at the > 250 m to 158,000 m scale. At this larger scale, the survey of North Carolina switchgrass found only a weak relationship between space and community composition [34]. Furthermore, we found that the importance of the within-site spatial scale depended on the niche. For instance, in a single site over a growing season, much of the variance in soil fungal community composition was explained by spatial distance from 0.1 to 250 m. In contrast, the majority of root community change seemingly occurred between 0.1 m and ~ 75 m (Fig. 3f).

We found that bacterial communities varied more between regional sampling sites compared to fungal communities. Bacterial communities’ greater responsiveness to large spatial drivers is consistent with recent global studies of soil microbial biogeography where spatial community turnover was much stronger for bacteria than for fungi [5]. Similar to previous studies on microbial community biogeography, we found that soil factors, specifically soil pH, were the dominant drivers of bacterial community structure [3]. Precipitation preceding sampling was also a consistent driver of bacterial communities suggesting that short-term precipitation patterns may partially underlay differences found in bacterial communities at regional scales [73]. Climate, especially precipitation, has also been found to be the major driver of global distributions of soil nematodes and earthworms [74, 75] which are major contributors to soil carbon cycling but beyond the scope of this study. Fungal communities were also strongly structured by soil pH, but plant traits, specifically root biomass and aboveground yield, were also significant drivers. Interestingly, a greenhouse bioassay study using soils from these same bioenergy land sites found that bacterial community structure was slightly more predictive of switchgrass productivity, compared to fungal community structure (variance explained: bacteria 45.0% versus fungi 38.9% [76]). Much of the spatial structuring in the fungal community was not explained by our measured factors suggesting possible dispersal limitations as has been predicted to be a dominant driver of soil fungal structure in other biogeographic studies [4, 12]. However, our sampling sites varied by other unaccounted for characteristics, such as land use history, the effects of which are difficult to model but may account for some of the unexplained variation at the site level.

Even though bacteria and fungi differentially responded to spatial factors, we found that there were strong correlations between these communities across sampling sites. Interestingly, the highest correlation between root bacterial and fungal communities occurred at Rhinelander which had the lowest microbial richness and levels of soil N and pH (Table S1). It is possible that at Rhinelander the root communities’ interactions are more apparent than at other sites due to the nutrient poor soils. Regardless of primary drivers, bacterial communities responded more strongly than fungal communities to the site-level conditions.

### Root microbial community change over a growing season

Over one growing season, at a single location (Lux Arbor), root bacterial community composition changed more temporally, compared to soil bacterial and fungal compositions which changed more spatially. Our results are consistent with a study that compared rhizosphere and soil bacterial community development within two growing seasons which found that rhizosphere communities show more turnover and increased network complexity compared to soils, as perennial crops grow [77]. In general, soil microbial communities have been found to show little temporal change and are more structured by soil depth [78], elevation [79], and space in general [73, 80–82]. Soil nematode communities have also been shown to undergo significant turnover during the growing season, but the magnitude of turnover may depend on site land use and soil pore size [83]. Interestingly, root fungal community change was only slightly more structured temporally than spatially (Table 3) and the similarity of the fungal root and soil communities was highest when the sampling day was paired temporally and spatially (Fig. 4b) suggesting more persistent linkages between roots and soils for fungal communities than for bacterial communities. It is also possible that the larger size of fungal individuals, compared to bacteria, would increase the likelihood of capturing fungal root symbionts in soils possibly leading to this higher spatiotemporal pairing. In bacterial communities, the temporal lag in the similarity between root and soil may suggest that roots are recruiting bacteria from the soil throughout the year, driving the significant temporal community structure in the root bacterial community.

It is also possible that the differential responses of the bacterial and fungal communities may be driven by the differences in generational times with bacterial communities likely more temporally responsive due to shorter generations. For example, a recent study of soil microbial turnover over the course of four years found that bacterial communities showed less community change than fungi [10]. Together, these indicate that a temporal scale of ‘years’ may better capture fungal turnover, while ‘growing season’ may better capture bacterial turnover.

Contrary to our hypothesis, plant traits and phenology were not drivers of the switchgrass microbiome temporal change but instead, weather conditions and soil factors were more consistently significant drivers. The only plant trait that we found as a significant predictor of community composition was subplot yield predicting the soil bacterial community. The yield was only measured once, at the end of the growing season, and the soil bacterial community was only weakly structured temporally, indicating that it is more likely an indicator of spatial community structure within Lux Arbor. On the other hand, the strongest predictor of the root fungal community was soil moisture measured at the soil core level. This soil moisture measurement captured both the spatial heterogeneity in water-holding capacity and temporal factors, such as precipitation and humidity. Our results indicate that the below-ground plant microbiome was not responsive to plant traits and phenology, as seasonality of the Great Lakes region outweighed plant host effects. It is possible that in ecosystems with less seasonality, host plants may be a more significant driver of microbiome composition. Interestingly, temporal distance remained in the final model of the root bacterial community, with no measured temporally explicit factors remaining, suggesting there is an unmeasured variable that is driving the temporal change in the root bacterial community. It is possible that soil bacteria recruitment into the root endosphere may be a partial driver of the root community change.

The greatest change in both root bacterial and fungal communities occurred toward the end of the growing season. This late season increase in the rate of community change corresponded with a reduced similarity between root and soil communities in both domains (Fig. 4) and lower correlations between root bacterial and fungal communities (Fig. S11ac). This divergence could be driven by the interactions between plant host senescence and seasonal changes in weather. For example, studies have captured a shift in the below-ground microbiome from a relatively stable symbiont-dominated community in early growing seasons, dependent on simple root exudated carbon, to a more active saprotrophic-dominated community, able to breakdown complex carbon sources, towards the end of growing seasons [84–86]. However, these shifts from root-associates to saprotrophs seem more consistently recorded for bacterial communities while fungal communities can lack the shifts in composition, suggesting that there are guild shifts where root-associated fungi become saprotrophs [87, 88]. These differential responses of the two domains to host senescence may underlay this difference in community trajectories. Although we did not measure this likely complex interaction, it may be important for understanding year to year differences in the seasonal dynamics of the switchgrass microbiome (unpublished data, [35])

### Core microbial community spatiotemporal consistency

Even though the switchgrass microbiome varied spatiotemporally, we identified a core community that persisted across space and time as a way to identify taxa that may consistently play important roles in switchgrass-dominated ecosystems. The most abundant species in the core root bacterial community, making up greater than 10% of reads, was a *Bradyrhizobium* sp., a group that has been identified as abundant members of the *nifH* community in this region and likely members of the free-living N fixing community associated with switchgrass [35, 89]. Additionally, Gammaproteobacteria were secondarily dominant taxa in our core community. The relative abundance of this class within the putative free-living N-fixing community (e.g., *nifH* community) has been positively correlated with high N fixation in switchgrass soils [35] and correlated with switchgrass aboveground productivity [76].

Although the core bacterial community appeared stable across the growing season, the core root fungal community was more dynamic, with the relative abundance of symbionts and saprotrophs dependent on the sampling site and collection date. The core soil fungal community was dominated across all sites and collection dates by possible saprotrophs and symbionts, Hypocreales, and Mortierellales. The root community was relatively more dynamic with the dominant core taxa depending on the site and collection date, and it is possible that this variation in the composition may have implications for host health. For example, Helotiales dominated Rhinelander while Auriculariales and saprotrophs dominated the core root communities of Escanaba. Helotiales are known to include dark-septate root endophyte taxa, some of which can benefit plant health, as their extracellular enzymes are known to be effective in obtaining organic nutrients from acidic soils (Jumpponen, 2001). Although many Auriculariales are known to fruit on dead wood, some genera such as *Oliveonia* may be associated with plant roots [90]. Additionally, the important guild of root mutualists, arbuscular mycorrhizal (AM) fungi, specifically those belonging to Glomerales and Paraglomerales, were dominant members within the core root community across sites and the growing season. Paraglomerales appear to be quite general and neutral in their effects on plants, but a bioassay study using soil from the sites we characterized in our study found that the abundance of Glomerales was predictive of switchgrass biomass [76].

Interestingly, the highest relative abundance of AM fungi was observed early in the growing season indicating an ecological strategy of quickly colonizing emerging fine roots. With the decline in the abundance of AM fungi towards the end of the growing season, other putative endophytes began to increase in relative abundance (Fig. S20a), suggesting that there may be phenological shifts in root symbionts.

### Weak effects of N on microbial communities

Nitrogen addition had weak effects on microbial community structure compared to the spatiotemporal factors. While we did find that N addition altered community composition and generally reduced diversity and richness in the soil and root microbiome, these effects depended on sites and collection dates. We also found that N addition increased root fungal diversity at our northern Michigan site and soil bacterial richness towards the end of the growing season suggesting that the effects of N addition are weak and inconsistent. Although nitrogen addition did significantly alter microbiome composition over the growing season, it explained < 3.2% of community variance (Table 2) and inorganic soil nitrogen was not a predictor of microbiome composition (Fig. S13 & S15). While many studies have documented changes in microbial communities with N addition [23], the strength of effect may depend on application rate, which was relatively low in our study (5.6 g/m^2^). A meta-analysis of the effects of N addition on soil bacteria in agroecosystems found that N reduced diversity, but the effect was only significant when the rate of application was greater than 10 g/m^2^ [22]. Furthermore, a study of switchgrass soil microbiomes found that excess N applied at a rate of 19.6 g/m^2^ altered the community composition, increased metabolic function, and reduced potential N_2_ fixation while N addition at a rate of 5.6 g/m^2^ had little to no effect on the soil microbial community [26, 32]. Our results, and results from other studies, suggest that the effects of N addition depend on application rates, but capturing weak effects of N addition may also depend on the timing of sampling and interactions with other abiotic factors such as soil phosphorus and climate.

The most apparent effect of N addition on community composition was the reduction in correlations between root bacterial and fungal communities towards the end of the growing season. It is likely that N addition alters the relative trajectory of the bacterial and fungal communities in switchgrass roots. However, under the single collection date used in our across-sampling-site analyses, switchgrass microbiome compositions, and correlations between communities, of Lux Arbor showed no response to N application. In support of the weak effects of N addition on the soil environment, a study of our bioenergy lands site found that N increased total organic and microbial nitrogen but had weak to no effect on all other measured soil variables [25]. Our sampling across a growing season did find a weak but significant effect of N on community compositions. In opposition to our hypothesis, the effects of N on community composition did not consistently peak after-N application. In an experiment near Lux Arbor, temporally explicit sampling of switchgrass monocultures found that N application of 5.6 g/m^2^ had no effect on soil bacteria and fungi across the four switchgrass growth stages sampled [26]. Our more temporally intensive sampling (six root and 15 soil collection dates) likely increased our ability to capture the weak effects of N. The effects of N addition were weak to non-existent and depended on site and collection date; therefore, we conclude that spatiotemporal variables were more important drivers of the switchgrass microbiome.

## Conclusion

In this study, we sampled a single variety of switchgrass in monoculture across a geographic region to understand factors that account for the spatiotemporal structure of root and soil microbiomes. We found that the switchgrass microbiome was structured both spatially and temporally and that the strength of these factors depended on the microbial domain and the niche that they inhabit. In general, bacterial communities were more spatiotemporally structured compared to fungal communities. The largest spatial scale of sampling across regional sites had the strongest effect on microbial communities, but all communities, except for soil fungi, were structured by time of sampling. The relative importance of spatiotemporal patterns in microbial communities also depended on the microbial niche. We found that root communities were more structured temporally and soil communities were more structured spatially. These differential responses of niches and domains to spatiotemporal factors likely affected plant microbiome interactions and assembly within the plant microbiome. We found fungal communities were spatiotemporally paired while root bacteria appeared to be continually recruited from the soil community leading to temporal lags in community similarity. Our study highlights the vast differences among drivers of below-ground microbiome composition and assembly between domains and niches across space and time.

## Electronic supplementary material

Below is the link to the electronic supplementary material.

Supplemental Tables: Table S1. Abiotic and biotic characteristics of switchgrass monocultures from the Marginal Land Experiment sites. Values are averages from core-level measurements, subplot-level measurements, and plot-level measurements. Values in parentheses represent the number of missing data points that were imputed before analyses. Table S2. Abiotic and biotic characteristics of switchgrass monocultures from each collection date at Lux Arbor across one growing season. Values are averages from core-level measurements, subplot-level measurements, and 24-hour average site-level measurements. Values in parentheses represent the number of missing data points that were imputed before analyses. Table S3. The number of Marginal Land Experiment microbiome samples that passed all quality filtering and were used in analyses. Table S4. The number of Lux Arbor microbiome samples from each collection date that passed all filtering and were used in analyses. Table S5. Post bioinformatics and post filtering species richness and read abundances for bacterial and fungal communities. Table S6. Combined guild categories of fungal species that received multiple guild classifications using FunGuild. Table S7. Results from mixed effects models testing the effects of site and nitrogen (N) addition on the residual error from Procrustes concordance between bacterial and fungal communities from roots and soils of switchgrass monocultures at the Marginal Land Experiment sites. Bolded texts highlight significant factors. Table S8. Results from mixed effects models testing the effects of site and nitrogen (N) addition on the richness and inverse Simpson diversity of bacterial and fungal communities from roots and soils of switchgrass monocultures at the Marginal Land Experiment sites. Bolded texts highlight significant factors. Table S9. Results from mixed effects models testing the effects of collection date and nitrogen (N) addition on the residual error from Procrustes concordance between bacterial and fungal communities from roots and soils of switchgrass monocultures across the growing season at Lux Arbor. Bolded texts highlight significant factors. Table S10. Results from mixed effects models testing the effects of collection date and nitrogen (N) addition on the richness and inverse Simpson diversity of bacterial and fungal communities from roots and soils of switchgrass monocultures across the growing season at Lux Arbor. Bolded texts highlight significant factors. Table S11. Results from mixed effects models testing the effects of site and nitrogen (N) addition on beta dispersion of bacterial and fungal communities from roots and soils of switchgrass monocultures at the Marginal Land Experiment sites. Bolded texts highlight significant factors. Table S12. Results from mixed effects models testing the effects of collection date, nitrogen (N) addition, and comparison date (soils either collected 2 weeks prior or same day as roots) on the similarity (1-Bray\ Curtis\ dist) of root bacterial and fungal communities to soil communities of switchgrass monocultures across the growing season at Lux Arbor. Bolded texts highlight significant factors. Table S13. Results from mixed effects models testing the effects of collection date and nitrogen (N) addition on beta dispersion of bacterial and fungal communities from roots and soils of switchgrass monocultures across the growing season at Lux Arbor. Bolded texts highlight significant factors. Table S14. Core bacterial and fungal community richness and relative abundance (percentage of reads) from roots and soils taken from switchgrass monocultures at the Marginal Land Experiment (MLE) sites and across the growing season at Lux Arbor. Figure S1. The fit of core bacterial communities in ab) roots and cd) soils of switchgrass monocultures across the Marginal Land Experiment sites. ac) Neutral model fits of abundance-occupancy curves of the bacterial communities with core members fitting the neutral model filled gray, core members with higher-than-expected frequency are filled purple, and core members at lower-than-expected frequency are filled black. All other members of the community are represented by unfilled symbols. bd) OTUs ranked by their abundance/occupancy versus the Bray-Curtis change with the exclusion of an OTU. The red line represents the last 5% increase in the Bray-Curtis distance. Figure S2. The fit of core fungal communities in ab) roots and cd) soils of switchgrass monocultures across the Marginal Land Experiment sites. a) Abundance-occupancy plot, neutral model failed to fit of the fungal communities with core members filled red and all other taxa are represented by unfilled symbols. c) Neutral model fits of abundance-occupancy curves of the fungal communities with core members fitting the neutral model filled gray, core members with higher-than-expected frequency are filled purple, and core members at lower-than-expected frequency are filled black. All other members of the community are represented by unfilled symbols. bd) OTUs ranked by their abundance/occupancy versus the Bray-Curtis change with the exclusion of an OTU. The red line represents the last 5% increase in the Bray-Curtis distance. Figure S3. The fit of core bacterial communities in ab) roots and cd) soils of switchgrass monocultures at Lux Arbor across the growing season. ac) Neutral model fits of abundance-occupancy curves of the bacterial communities with core members fitting the neutral model filled gray, core members with higher-than-expected frequency are filled purple, and core members at lower-than-expected frequency are filled black. All other members of the community are represented by unfilled symbols. bd) OTUs ranked by their abundance/occupancy versus the Bray-Curtis change with the exclusion of an OTU. The red line represents the last 5% increase in the Bray-Curtis distance. Figure S4. The fit of core fungal communities in ab) roots and cd) soils of switchgrass monocultures at Lux Arbor across the growing season. ac) Neutral model fits of abundance-occupancy curves of the fungal communities with core members fitting the neutral model filled gray, core members with higher-than-expected frequency are filled purple, and core members at lower-than-expected frequency are filled black. All other members of the community are represented by unfilled symbols. bd) OTUs ranked by their abundance/occupancy versus the Bray-Curtis change with the exclusion of an OTU. The red line represents the last 5% increase in the Bray-Curtis distance. Figure S5. The proportion of ac) richness and bd) reads included in the core bacterial community at the Bray-Curtis cut-offs for ab) roots and cd) soils of switchgrass monocultures of the Marginal Land Experiment. The red line represents the chosen threshold of a 5% increase in Bray-Curtis. Figure S6. The proportion of ac) richness and bd) reads included in the core fungal community at the Bray-Curtis cut-offs for ab) roots and cd) soils of switchgrass monocultures of the Marginal Land Experiment. The red line represents the chosen threshold of a 5% increase in Bray-Curtis. Figure S7. The proportion of ac) richness and bd) reads included in the core bacterial community at the Bray-Curtis cut-offs for ab) roots and cd) soils of switchgrass monocultures across one growing season at Lux Arbor. The red line represents the chosen threshold of a 5% increase in Bray-Curtis. Figure S8. The proportion of ac) richness and bd) reads included in the core fungal community at the Bray-Curtis cut-offs for ab) roots and cd) soils of switchgrass monocultures across one growing season at Lux Arbor. The red line represents the chosen threshold of a 5% increase in Bray-Curtis. Figure S9. NMDS plots with Procrustes rotations of the fungal communities onto bacterial communities of a) root and b) soils of switchgrass monocultures at the Marginal Land Experiment in nitrogen (N) addition (filled circles) and control (open triangles) subplots sampled July 2018. Boxplot of the residual errors from the Procrustes models for c) roots and d) soils in N addition (dark fill) and control (light fill) subplots. Lowercase letters represent significant differences between sites for roots. Uppercase letters represent significant differences between sites for soil. “***” represents p < 0.001 Tukey HSD adjusted significance for the effect of N addition. Figure S10. a) Bacterial richness, b) fungal richness, c) bacterial diversity, and d) fungal diversity (inverse Simpson) of switchgrass monocultures at each Marginal Land Experiment site from nitrogen (N) addition (dark fill) and control (light fill) subplots. Lowercase letters represent significant differences between sites for roots. Uppercase letters represent significant differences between sites for soil. “#”, “**”, and “***” represents p < 0.10, p < 0.01, and p < 0.001 Tukey HSD adjusted significance for the effect of N addition. Figure S11. NMDS plots with Procrustes rotations of the fungal communities onto bacterial communities of a) root and b) soils of switchgrass monocultures at Lux Arbor from across one growing season in nitrogen (N) addition (filled circles) and control (open triangles) subplots. Boxplot of the residual errors from the Procrustes models for c) roots and d) soils from N addition (dark fill) and control (light fill) subplots. Fill color ramp represents collection dates with lighter colors representing earlier dates and darker colors later dates. “#”, “*”, and “**” represents p < 0.10, p < 0.05, and p < 0.01 Tukey HSD adjusted significance for the effect of N addition. Figure S12. a) Bacterial richness, b) fungal richness, c) bacterial diversity, and d) fungal diversity (inverse Simpson) of switchgrass monocultures across one growing season at Lux Arbor in nitrogen (N) addition (dark fill) and control (light fill) subplots. Fill color ramp represents collection dates with lighter colors representing earlier dates and darker colors representing later dates. “#”, “*”, and “**” represents p < 0.10, p < 0.05, and p < 0.01 Tukey HSD adjusted significance for the effect of N addition. Figure S13. Loess graphs from generalized dissimilarity models (GDMs) of the a) root bacterial, b) root fungal, c) soil bacterial, and d) soil fungal communities of switchgrass monocultures from sites of the Marginal Land Experiment. Variables were scaled (0 to 1) to allow for comparison. Panel text represents the significance and percentage of deviance explained by GDMs. Figure S14. Beta dispersion in a) bacterial and b) fungal communities of the roots and soils of switchgrass monocultures at the Marginal Land Experiment sites from nitrogen (N) addition (dark fill) and control (light fill) subplots. Lowercase letters represent significant differences between sites for roots. Uppercase letters represent significant differences between sites for soil. Nitrogen addition did not have a significant effect on beta dispersion. Figure S15. Loess graphs from generalized dissimilarity models (GDMs) of the a) root bacterial, b) root fungal, c) soil bacterial, and d) soil fungal communities of switchgrass monocultures from Lux Arbor across one growing season. Variables were scaled (0 to 1) to allow for comparison. Panel text represents the significance and percentage of deviance explained by GDMs. Figure S16. Beta dispersion in a) bacterial and b) fungal communities of the roots and soils of switchgrass monocultures at Lux Arbor from N addition (dark fill) and control (light fill) subplots across one growing season. Fill color ramp represents collection dates with lighter colors representing earlier dates and darker colors later dates. Nitrogen addition did not have a significant effect on beta dispersion. Figure S17. Heat phylogenetic trees of families within core a) root bacterial, b) root fungal, c) soil bacterial, and d) soil fungal communities of switchgrass monocultures from sites of the Marginal Land Experiment. Node size represents richness (# of OTUs) and node color represents the log2 ratio of median proportions of read abundance between root (green colors) or soil (brown colors) communities. Grey nodes represent non-significant differences in taxa abundance (p > 0.05; false discovery rate adjusted Wilcoxon rank-sum pairwise test). Figure S18. Heat phylogenetic tree of families within core a) root bacterial, b) root fungal, c) soil bacterial, and d) soil fungal communities of switchgrass monocultures from Lux Arbor across one growing season. Node size represents richness (# of OTUs) and node color represents the log2 ratio of median proportions of read abundance between root (green colors) or soil (brown colors) communities. Grey nodes represent non-significant differences in taxa abundance (p > 0.05; false discovery rate adjusted Wilcoxon rank-sum pairwise test). Figure S19. Stacked bar graphs of the FunGuild classifications of core a) root and b) soil fungal communities of switchgrass monocultures at sites of the Marginal Land Experiment. Guilds with multiple hits are grouped. Figure S20. Stacked bar graphs of the FunGuild classifications of core a) root and b) soil fungal communities of switchgrass monocultures from Lux Arbor across one growing season. Guilds with multiple hits are grouped.

Supplementary Material 2

## Data Availability

Sequence data were deposited in the NCBI Short Read Archive under BioProject PRJNA733764 for bacterial communities and PRJNA799201 for fungal communities. All code for analyses and figures is available at DOI: 10.5281/zenodo.7307179 and processed data is available at OFS DOI: 10.17605/OSF.IO/5VW9C.
