# Supplementary material for: Regional biogeography versus intra-annual dynamics of the root and soil microbiome
Source: Environ Microbiome. 2023 Jun 7;18:50. doi: 10.1186/s40793-023-00504-x (PMC10245661; doi:10.1186/s40793-023-00504-x)
Supplement: Supplementary file 1 — Supplemental Tables: Table S1. Abiotic and biotic characteristics of switchgrass monocultures from the Marginal Land Experiment sites. Values are averages from core-level measurements, subplot-level measurements, and plot-level measurements. Values in parentheses represent the number of missing data points that were imputed before analyses. Table S2. Abiotic and biotic characteristics of switchgrass monocultures from each collection date at Lux Arbor across one growing season. Values are averages from core-level measurements, subplot-level measurements, and 24-hour average site-level measurements. Values in parentheses represent the number of missing data points that were imputed before analyses. Table S3. The number of Marginal Land Experiment microbiome samples that passed all quality filtering and were used in analyses. Table S4. The number of Lux Arbor microbiome samples from each collection date that passed all filtering and were used in analyses. Table S5. Post bioinformatics and post filtering species richness and read abundances for bacterial and fungal communities. Table S6. Combined guild categories of fungal species that received multiple guild classifications using FunGuild. Table S7. Results from mixed effects models testing the effects of site and nitrogen (N) addition on the residual error from Procrustes concordance between bacterial and fungal communities from roots and soils of switchgrass monocultures at the Marginal Land Experiment sites. Bolded texts highlight significant factors. Table S8. Results from mixed effects models testing the effects of site and nitrogen (N) addition on the richness and inverse Simpson diversity of bacterial and fungal communities from roots and soils of switchgrass monocultures at the Marginal Land Experiment sites. Bolded texts highlight significant factors. Table S9. Results from mixed effects models testing the effects of collection date and nitrogen (N) addition on the residual error from Procrustes concordance be [file 40793_2023_504_MOESM1_ESM.docx]

**SUPPLEMENTARY MATERIAL**

**Regional biogeography versus intra-annual dynamics of the root and soil microbiome**

Lukas P. Bell-Dereske, Gian Maria Niccolò Benucci, Pedro Beschoren da Costa, Gregory Bonito, Maren L. Friesen, Lisa K. Tiemann, and Sarah E. Evans

Supplemental Tables pages 1-14

Supplemental Figures pages 15-34

**SUPPLEMENTAL** **TABLES**

Table S1. Abiotic and biotic characteristics of switchgrass monocultures from the Marginal Land Experiment sites. Values are averages from core-level measurements, subplot-level measurements, and plot-level measurements. Values in parentheses represent the number of missing data points that were imputed before analyses.

|  |  |  |  | Soil core measurements^2^ | | | | | | | Subplot measurements^2^ | |  | Plot measurements^5^ | | | |
| --- | --- | --- | --- | --- | --- | --- | --- | --- | --- | --- | --- | --- | --- | --- | --- | --- | --- |
|  | Coords.  (Lat., Long.) | Soil classification^1^ | Date | Shoot mass (g) | Soil NH_4_ (µg/g) | Soil NO_3_ (µg/g) | Soil TOC (mg/kg) | Soil TON (mg/kg) | Ratio Soil TOC:TON | Soil moist. (%) | Root mass^2^ (g) | Subplot yield^3^ (kg/m^2^) | 7-day rain^3^ (mm) | Soil pH | Soil K (ppm) | Soil Ca (ppm) | Soil P (ppm)^6^ |
| Lux Arbor | 42.4763,  -85.4518 | Typic Hapludalf (Alfisol) loam | July 30, 2018 | 8.43 | 1.68 | 1.18 | 77.88 | 14.03 | 5.61 | 12.41 (1) | 0.19  (2) | 0.610 | 2.79 | 5.74 | 54.63 | 790.13 | 24.25 |
| Lake City | 44.3095,  -85.2032 | Oxyaquic Haplorthod (Spodosol) sandy loam | July 10, 2018 | 4.06 | 1.60 | 1.08  (2) | 102.00  (1) | 17.41 | 5.89  (1) | 6.53 | 0.17 | 0.712 | 14.73 | 6.70 | 30.38 | 723.38 | 44.25 |
| Escanaba | 45.7646,  -87.1838 | Inceptic Hapludalf (Alfisol) sandy loam | July 24, 2018 | 5.65 | 2.66  (2) | 0.32 | 142.98  (1) | 20.80 | 7.15  (1) | 18.68 | 0.16 | 0.949 | 8.13 | 6.89 | 43.13 | 1326.50 | 9.50 |
| Hancock | 44.1191,  -89.5332 | Typic Udipsamment (Entisol) loamy sand | July 16, 2018 | 3.92 | 1.16 | 1.38  (2) | 74.27 | 12.74 | 5.78 | 3.65 | 0.12 | 0.330 | 2.29 | 6.22 | 61.67 | 536.83 | 72.00 |
| Rhinelander | 45.6668,  -89.2186 | Entic Haplorthod (Spodosol) sandy loam | July 23, 2018 | 4.59 | 0.59 | 0.11 | 163.31  (3) | 13.57 | 11.61  (5) | 14.86 | 0.14 | 0.618 | 38.10 | 5.46 | 77.63 | 383.75 | 281.38 |

^1^Kasmerchak, C. S., and R. Schaetzl. 2018. Soils of the GLBRC Marginal Land Experiment (MLE) Sites. Kellogg Biological Station Long-Term

Ecological Research Special Publication.

^2^see methods in Smercina, D. N., S. E. Evans, M. L. Friesen, and L. K. Tiemann. 2021. Temporal dynamics of free-living nitrogen fixation in the switchgrass rhizosphere. GCB Bioenergy 13:1814–1830.

^3^https://data.sustainability.glbrc.org/datatables/465

^4^https://data.sustainability.glbrc.org/datatables/507

^5^https://data.sustainability.glbrc.org/datatables/438

^6^Not used in the generalized dissimilarity models because of correlation with soil ammonium (Pearson’s *r* = -0.792). Retained here for reference.

Table S2. Abiotic and biotic characteristics of switchgrass monocultures from each collection date at Lux Arbor across one growing season. Values are averages from core-level measurements, subplot-level measurements, and 24-hour average site-level measurements. Values in parentheses represent the number of missing data points that were imputed before analyses.

|  | Soil core measurements^1^ | | | | | | | | Subplot average measurements^1^ | 24-hr site measurements^3^ | |
| --- | --- | --- | --- | --- | --- | --- | --- | --- | --- | --- | --- |
| Date | Soil NH_4_ (µg/g) | Soil NO_3_ (µg/g) | Soil TOC (µg/g) | Soil TON (µg/g) | Ratio Soil TOC:TON | Shoot mass (g) | SLA | Soil moist. (%) | Root mass (g) | Soil temp. (°C) | 7-day rain (mm) |
| April 30, 2018 | 1.98 | 0.27 | 174.81  (2) | 25.93 | 6.80  (2) | not emerged | not emerged | 16.01 | 0.31  (1) | 9.50 | 0.51 |
| May 15, 2018 | 2.21  (11) | 0.83  (1) | 129.48  (3) | 27.80  (1) | 4.99  (4) | 0.17 | 128.41 | 23.06 | 0.26 | 16.10 | 87.12 |
| May 29, 2018 | 1.82 | 0.77 | 79.06 | 15.75 | 5.08 | 0.92 | 126.66  (1) | 13.86 | 0.11  (1) | 21.28 | 1.78 |
| June 11, 2018 | 1.47 | 0.08 | 174.57  (9) | 19.43 | 9.13  (9) | 2.85 | 144.64 | 21.88 | 0.10  (1) | 18.55 | 44.71 |
| June 25, 2018 | 1.82 | 0.36 | 119.87  (5) | 19.95 | 6.81  (5) | 4.54 | 158.70 | 20.90 | 0.11 | 18.66 | 30.23 |
| July 9, 2018 | 1.77 | 0.24 | 116.93 | 14.37 | 7.64  (1) | 6.66 | 142.55 | 13.77 | 0.13  (1) | 19.96 | 4.06 |
| July 30, 2018 | 1.68 | 1.18 | 77.88 | 14.03 | 5.61 | 8.43 | 134.38 | 12.41  (1) | 0.19  (2) | 19.09 | 2.79 |
| August 8, 2018 | 1.35 | 0.75 | 77.41 | 11.22 | 6.91  (1) | 8.16 | 141.13 | 20.24 | 0.13 | 21.20 | 39.12 |
| August 20, 2018 | 1.36 | 1.15  (1) | 92.68 | 8.73 | 10.97  (3) | 7.21  (1) | 142.42 | 11.52 | 0.13  (1) | 20.94 | 0.51 |
| September 4, 2018 | 1.58 | 0.73 | 83.69 | 15.15 | 5.65 | 9.21 | 128.17 | 20.50 | 0.19  (3) | 21.72 | 138.93 |
| September 17, 2018 | 1.03 | 0.50 | 62.86 | 8.85 | 8.09 | 8.18  (1) | 134.50 | 16.40 | 0.14 | 19.56 | 0.00 |
| October 3, 2018 | 1.41 | 0.26 | 84.75 | 15.34 | 5.62 | 6.61 | 137.80 | 20.42  (1) | 0.10  (1) | 15.53 | 38.61 |
| October 15, 2018 | 1.38 | 0.38 | 109.65  (2) | 15.19 | 7.34  (3) | 7.42 | 132.61 | 22.26 | 0.21  (1) | 12.13 | 34.55 |
| November 5, 2018 | 1.96 | 0.32 | 104.75  (1) | 16.78 | 6.58  (1) | harvested | harvested | 21.95 | 0.14  (2) | 8.31 | 5.59 |

^1^see methods in Smercina, D. N., S. E. Evans, M. L. Friesen, and L. K. Tiemann. 2021. Temporal dynamics of free-living nitrogen fixation in the switchgrass rhizosphere. GCB Bioenergy 13:1814–1830.

^2^https://data.sustainability.glbrc.org/datatables/507

Table S3. The number of Marginal Land Experiment microbiome samples that passed all quality filtering and were used in analyses.

|  | No. of replicates | Root bacteria | Root fungi | Soil bacteria | Soil fungi |
| --- | --- | --- | --- | --- | --- |
| Lux Arbor | 4 | 23 | 24 | 24 | 23 |
| Lake City | 4 | 24 | 22 | 24 | 24 |
| Escanaba | 4 | 24 | 23 | 24 | 24 |
| Hancock | 3 | 18 | 18 | 18 | 18 |
| Rhinelander | 4 | 24 | 24 | 24 | 23 |

Table S4. The number of Lux Arbor microbiome samples from each collection date that passed all filtering and were used in analyses.

| Collection date | Root bacteria | Root fungi | Soil bacteria | Soil fungi |
| --- | --- | --- | --- | --- |
| March 19, 2018 |  |  | 24 | 22 |
| April 30, 2018 |  |  | 23 | 24 |
| May 15, 2018 |  |  | 24 | 24 |
| May 29, 2018 | 24 | 22 | 23 | 24 |
| June 11, 2018 |  |  | 24 | 24 |
| June 25, 2018 | 24 | 24 | 24 | 22 |
| July 9, 2018 |  |  | 24 | 24 |
| July 30, 2018 | 23 | 24 | 24 | 23 |
| August 8, 2018 |  |  | 24 | 21 |
| August 20, 2018 | 24 | 24 | 22 | 24 |
| September 4, 2018 |  |  | 22 | 24 |
| September 17, 2018 | 24 | 23 | 24 | 23 |
| October 3, 2018 | 23 | 24 | 23 | 24 |
| October 15, 2018 |  |  | 24 | 22 |
| November 5, 2018 |  |  | 24 | 24 |

Table S5. Post bioinformatics and post filtering species richness and read abundances for bacterial and fungal communities.

|  |  | Total richness | Root Samples | Root richness | Root read abundance | Soil samples | Soil richness | Soil read abundance |
| --- | --- | --- | --- | --- | --- | --- | --- | --- |
| Post bioinformatics | Bacteria | 46,923 | 234 | 27,005 | 11,536,151 | 870 | 45,633 | 20,408,369 |
|  | Fungi | 6,871 | 234 | 5,963 | 14,818,135 | 865 | 6,858 | 90,388,075 |
| Post filtering and rarefying (10,000 reads) | Bacteria | 31,837 | 233 | 17,499 | 2,330,000 | 859 | 31,361 | 8,590,000 |
|  | Fungi | 3,088 | 228 | 2,604 | 2,280,000 | 846 | 3,072 | 8,460,000 |

Table S6. Combined guild categories of fungal species that received multiple guild classifications using FunGuild^1^.

| Simplified Guild | Original Guilds |
| --- | --- |
| Multiple Saprotroph | Plant Saprotroph-Wood Saprotroph  Soil Saprotroph-Undefined Saprotroph  Dung Saprotroph-Undefined Saprotroph |
| Symbiotroph-Saprotroph | Endophyte-Litter Saprotroph-Soil Saprotroph-Undefined Saprotroph |
| Symbiotroph-Pathogen | Endophyte-Plant Pathogen |
| Pathogen-Saprotroph | Animal Pathogen-Fungal Parasite-Undefined Saprotroph  Plant Pathogen-Wood Saprotroph  Fungal Parasite-Plant Pathogen-Plant Saprotroph |
| Symbiotroph-Pathogen-Saprotroph | Animal Pathogen-Endophyte-Epiphyte-Fungal Parasite-Plant Pathogen-Wood Saprotroph  Ectomycorrhizal-Fungal Parasite-Plant Pathogen-Wood Saprotroph |

^1^Nguyen NH, Song Z, Bates ST, Branco S, Tedersoo L, Menke J, et al. FUNGuild: An open annotation tool for parsing fungal community datasets by ecological guild. Fungal Ecology. 2016;20:241-8; doi: https://doi.org/10.1016/j.funeco.2015.06.006.

Table S7. Results from mixed effects models testing the effects of site and nitrogen (N) addition on the residual error from Procrustes concordance between bacterial and fungal communities from roots and soils of switchgrass monocultures at the Marginal Land Experiment sites. Bolded texts highlight significant factors.

|  | Root | | Soil | |
| --- | --- | --- | --- | --- |
|  | F-stat | P-value | F-stat | P-value |
| Site | **27.03** | **<0.001** | **22.42** | **<0.001** |
| N add | 1.69 | 0.197 | 0.98 | 0.326 |
| Site*N add | **3.29** | **0.014** | 1.84 | 0.126 |

Table S8. Results from mixed effects models testing the effects of site and nitrogen (N) addition on the richness and inverse Simpson diversity of bacterial and fungal communities from roots and soils of switchgrass monocultures at the Marginal Land Experiment sites. Bolded texts highlight significant factors.

|  | Observed Richness | | | | Inverse Simpson | | | |
| --- | --- | --- | --- | --- | --- | --- | --- | --- |
|  | Root Bacteria | | Soil Bacteria | | Root Bacteria | | Soil Bacteria | |
|  | F-stat | P-value | F-stat | P-value | F-stat | P-value | F-stat | P-value |
| Site | **40.59** | **<0.001** | **33.24** | **<0.001** | **15.61** | **<0.001** | **7.96** | **<0.001** |
| N add | **16.42** | **<0.001** | 1.63 | 0.204 | **11.64** | **0.001** | 1.51 | 0.222 |
| Site*N add | **3.06** | **0.020** | 0.26 | 0.906 | 1.38 | 0.245 | 0.43 | 0.790 |
|  | Root Fungi | | Soil Fungi | | Root Fungi | | Soil Fungi | |
|  | F-stat | P-value | F-stat | P-value | F-stat | P-value | F-stat | P-value |
| Site | **9.43** | **<0.001** | **29.88** | **<0.001** | **2.77** | **0.031** | **3.24** | **0.015** |
| N add | 0.79 | 0.377 | 2.33 | 0.130 | 0.60 | 0.442 | 0.03 | 0.864 |
| Site*N add | 1.82 | 0.132 | 0.57 | 0.682 | **4.07** | **0.004** | 1.88 | 0.120 |

Table S9. Results from mixed effects models testing the effects of collection date and nitrogen (N) addition on the residual error from Procrustes concordance between bacterial and fungal communities from roots and soils of switchgrass monocultures across the growing season at Lux Arbor. Bolded texts highlight significant factors.

|  | Root | | Soil | |
| --- | --- | --- | --- | --- |
|  | F-stat | P-value | F-stat | P-value |
| Collection Date | **2.43** | **0.038** | 1.07 | 0.383 |
| N add | **11.93** | **0.001** | **4.51** | **0.035** |
| Date*N add | **3.02** | **0.013** | 0.74 | 0.734 |

Table S10. Results from mixed effects models testing the effects of collection date and nitrogen (N) addition on the richness and inverse Simpson diversity of bacterial and fungal communities from roots and soils of switchgrass monocultures across the growing season at Lux Arbor. Bolded texts highlight significant factors.

|  | Observed Richness | | | | Inverse Simpson | | | |
| --- | --- | --- | --- | --- | --- | --- | --- | --- |
|  | Root Bacteria | | Soil Bacteria | | Root Bacteria | | Soil Bacteria | |
|  | F-stat | P-value | F-stat | P-value | F-stat | P-value | F-stat | P-value |
| Collection Date | **5.44** | **<0.001** | **17.65** | **<0.001** | **9.17** | **<0.001** | **5.15** | **<0.001** |
| N add | 2.42 | 0.122 | **4.11** | **0.043** | 2.56 | 0.112 | **3.93** | **0.048** |
| Date*N add | 1.24 | 0.293 | 1.46 | 0.117 | 0.54 | 0.743 | 0.87 | 0.589 |
|  | Root Fungi | | Soil Fungi | | Root Fungi | | Soil Fungi | |
|  | F-stat | P-value | F-stat | P-value | F-stat | P-value | F-stat | P-value |
| Collection Date | **23.55** | **<0.001** | **8.57** | **<0.001** | **7.95** | **<0.001** | 1.69 | 0.057 |
| N add | 3.58 | 0.061 | 0.50 | 0.481 | **10.92** | **0.001** | 0.67 | 0.414 |
| Date*N add | 0.88 | 0.499 | 1.02 | 0.428 | 0.65 | 0.662 | 0.97 | 0.483 |

Table S11. Results from mixed effects models testing the effects of site and nitrogen (N) addition on beta dispersion of bacterial and fungal communities from roots and soils of switchgrass monocultures at the Marginal Land Experiment sites. Bolded texts highlight significant factors.

|  | Root Bacteria | | Root Fungi | |
| --- | --- | --- | --- | --- |
|  | F-stat | P-value | F-stat | P-value |
| Site | **6.31** | **<0.001** | **6.55** | **<0.001** |
| N add | 1.07 | 0.304 | 0.95 | 0.333 |
| Site*N add | 1.03 | 0.396 | 0.22 | 0.927 |
|  | Soil Bacteria | | Soil Fungi | |
|  | F-stat | P-value | F-stat | P-value |
| Site | **28.79** | **<0.001** | **17.51** | **<0.001** |
| N add | 1.03 | 0.313 | 1.33 | 0.252 |
| Site*N add | 0.26 | 0.906 | 1.23 | 0.302 |

Table S12. Results from mixed effects models testing the effects of collection date, nitrogen (N) addition, and comparison date (soils either collected 2 weeks prior or same day as roots) on the similarity ($1-Bray Curtis dist$) of root bacterial and fungal communities to soil communities of switchgrass monocultures across the growing season at Lux Arbor. Bolded texts highlight significant factors.

|  | Bacteria | | Fungi | |
| --- | --- | --- | --- | --- |
|  | F-stat | P-value | F-stat | P-value |
| Collection Date | **9.12** | **<0.001** | **10.74** | **<0.001** |
| N add | 0.22 | 0.639 | 1.75 | 0.188 |
| Comparison date | **25.19** | **<0.001** | **48.41** | **<0.001** |
| Date*N add | 0.75 | 0.589 | 0.73 | 0.605 |
| Date*Comp | **5.45** | **<0.001** | 1.47 | 0.200 |
| N add*Comp | 0.01 | 0.930 | 1.03 | 0.310 |
| Date*N add*Comp | 0.79 | 0.559 | 0.73 | 0.600 |

Table S13. Results from mixed effects models testing the effects of collection date and nitrogen (N) addition on beta dispersion of bacterial and fungal communities from roots and soils of switchgrass monocultures across the growing season at Lux Arbor. Bolded texts highlight significant factors.

|  | Root Bacteria | | Root Fungi | |
| --- | --- | --- | --- | --- |
|  | F-stat | P-value | F-stat | P-value |
| Collection Date | **2.95** | **0.015** | **2.55** | **0.031** |
| N add | <0.01 | 0.970 | 1.91 | 0.169 |
| Date*N add | 1.18 | 0.321 | 0.39 | 0.858 |
|  | Soil Bacteria | | Soil Fungi | |
|  | F-stat | P-value | F-stat | P-value |
| Collection Date | **2.84** | **<0.001** | **2.20** | **0.008** |
| N add | 2.96 | 0.086 | 1.99 | 0.160 |
| Date*N add | 1.09 | 0.369 | 0.57 | 0.886 |

Table S14. Core bacterial and fungal community richness and relative abundance (percentage of reads) from roots and soils taken from switchgrass monocultures at the Marginal Land Experiment (MLE) sites and across the growing season at Lux Arbor.

|  | MLE core richness | MLE percentage of reads | Lux Arbor  core richness | Lux Arbor  percentage of reads |
| --- | --- | --- | --- | --- |
| Root Bacteria | 33 | 34.2% | 66 | 45.6% |
| Soil Bacteria | 133 | 30.0% | 74 | 26.7% |
| Root Fungi | 123 | 59.9% | 60 | 66.0% |
| Soil Fungi | 60 | 46.7% | 154 | 69.7% |

**SUPPLEMENTAL** **FIGURES**


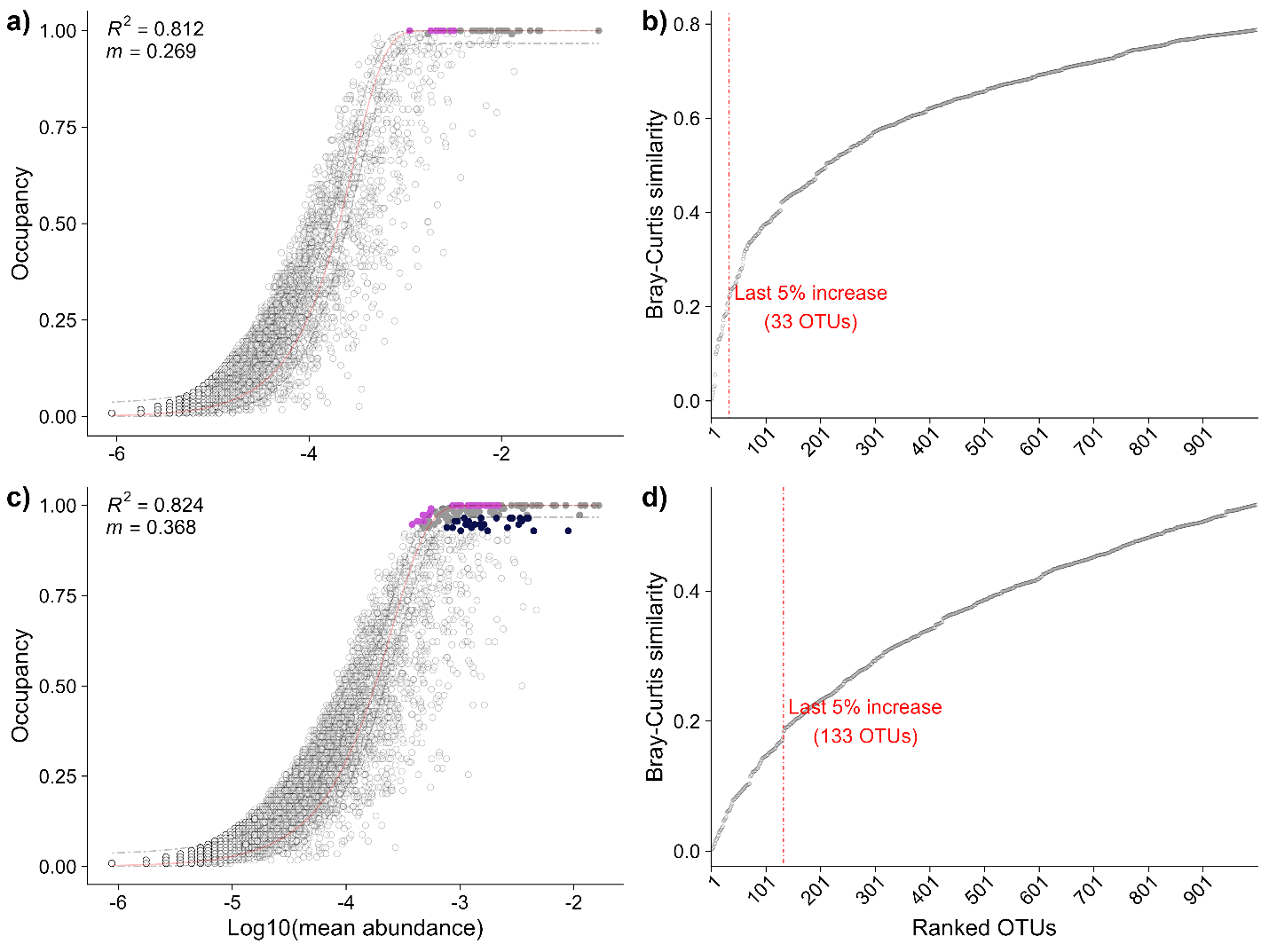
Figure S1. The fit of core bacterial communities in ab) roots and cd) soils of switchgrass monocultures across the Marginal Land Experiment sites. ac) Neutral model fits of abundance-occupancy curves of the bacterial communities with core members fitting the neutral model filled gray, core members with higher-than-expected frequency are filled purple, and core members at lower-than-expected frequency are filled black. All other members of the community are represented by unfilled symbols. bd) OTUs ranked by their abundance/occupancy versus the Bray-Curtis change with the exclusion of an OTU. The red line represents the last 5% increase in the Bray-Curtis distance.


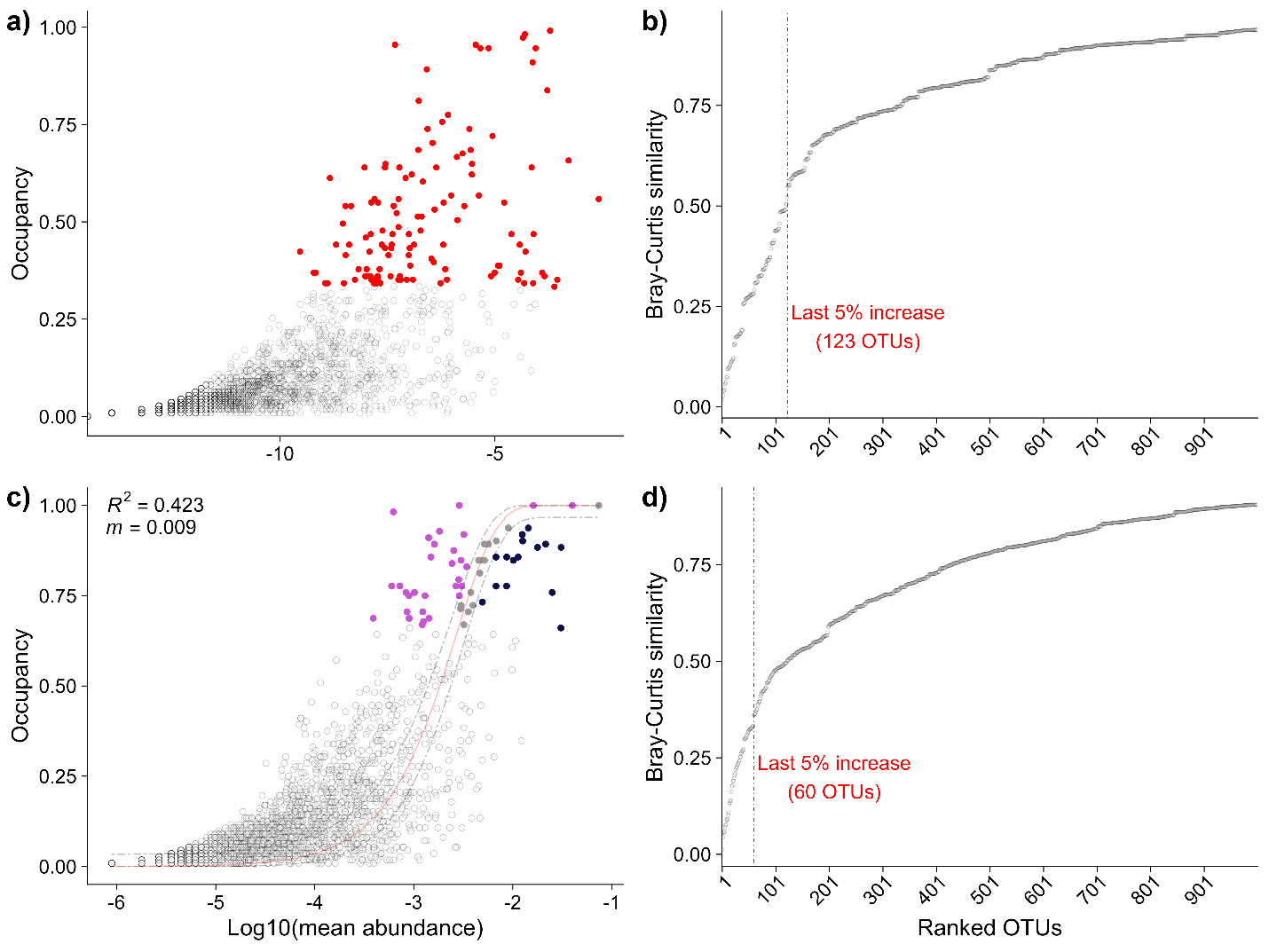


Figure S2. The fit of core fungal communities in ab) roots and cd) soils of switchgrass monocultures across the Marginal Land Experiment sites. a) Abundance-occupancy plot, neutral model failed to fit, of the fungal communities with core members filled red and all other taxa are represented by unfilled symbols. c) Neutral model fits of abundance-occupancy curves of the fungal communities with core members fitting the neutral model filled gray, core members with higher-than-expected frequency are filled purple, and core members at lower-than-expected frequency are filled black. All other members of the community are represented by unfilled symbols. bd) OTUs ranked by their abundance/occupancy versus the Bray-Curtis change with the exclusion of an OTU. The red line represents the last 5% increase in the Bray-Curtis distance.





Figure S3. The fit of core bacterial communities in ab) roots and cd) soils of switchgrass monocultures at Lux Arbor across the growing season. ac) Neutral model fits of abundance-occupancy curves of the bacterial communities with core members fitting the neutral model filled gray, core members with higher-than-expected frequency are filled purple, and core members at lower-than-expected frequency are filled black. All other members of the community are represented by unfilled symbols. bd) OTUs ranked by their abundance/occupancy versus the Bray-Curtis change with the exclusion of an OTU. The red line represents the last 5% increase in the Bray-Curtis distance.


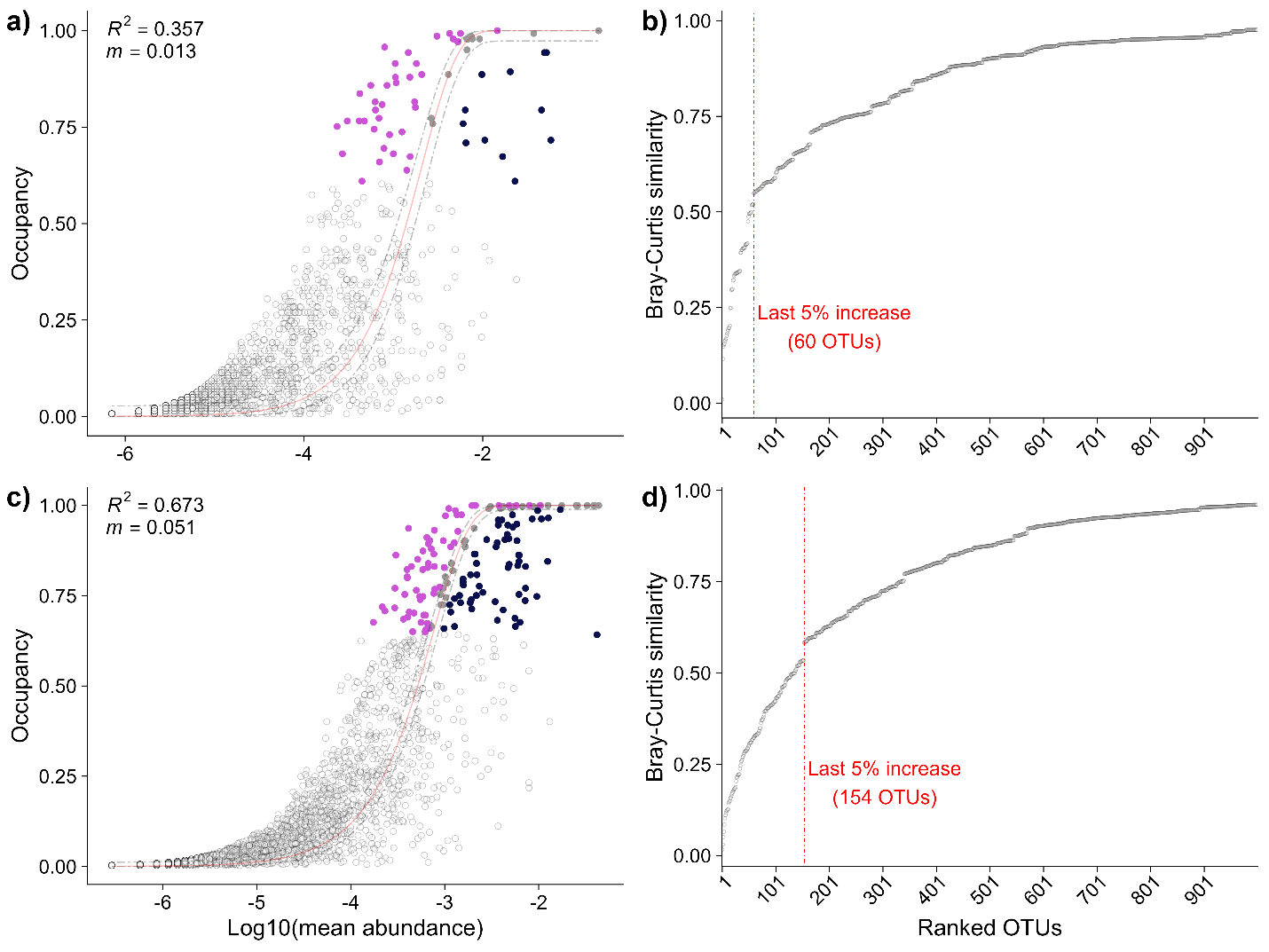


Figure S4. The fit of core fungal communities in ab) roots and cd) soils of switchgrass monocultures at Lux Arbor across the growing season. ac) Neutral model fits of abundance-occupancy curves of the fungal communities with core members fitting the neutral model filled gray, core members with higher-than-expected frequency are filled purple, and core members at lower-than-expected frequency are filled black. All other members of the community are represented by unfilled symbols. bd) OTUs ranked by their abundance/occupancy versus the Bray-Curtis change with the exclusion of an OTU. The red line represents the last 5% increase in the Bray-Curtis distance.


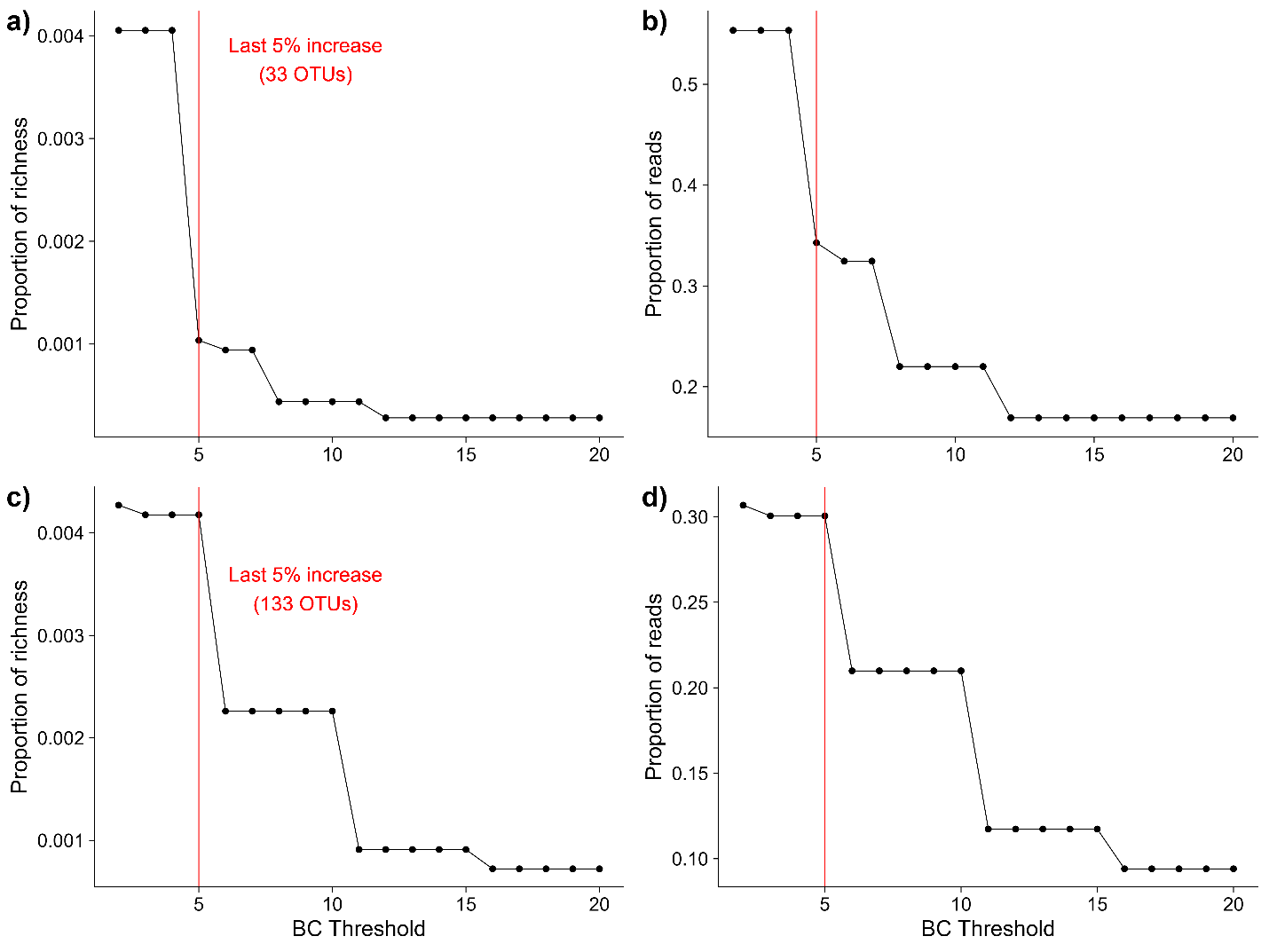


Figure S5. The proportion of ac) richness and bd) reads included in the core bacterial community at the Bray-Curtis cut-offs for ab) roots and cd) soils of switchgrass monocultures of the Marginal Land Experiment. The red line represents the chosen threshold of a 5% increase in Bray-Curtis.


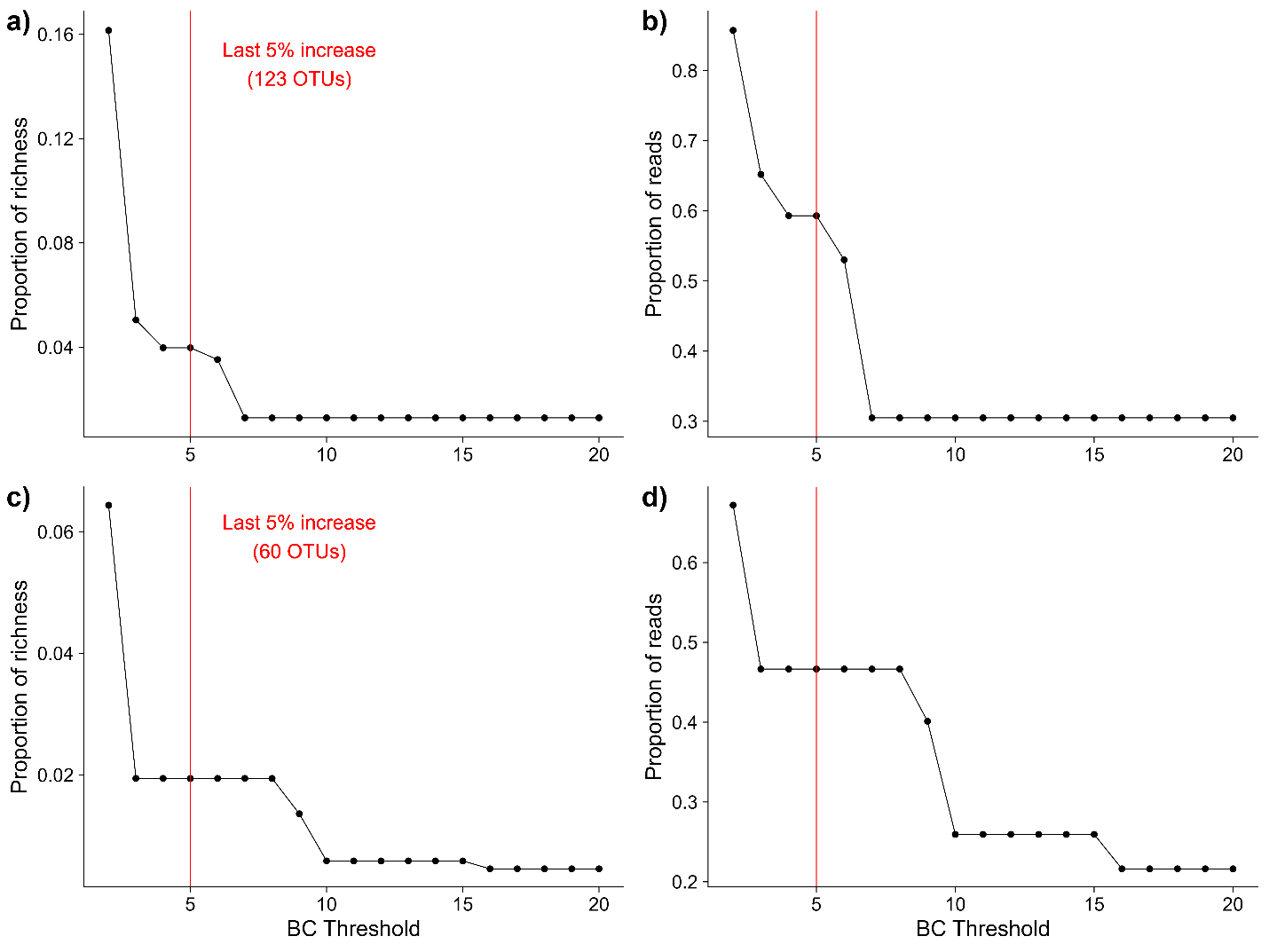


Figure S6. The proportion of ac) richness and bd) reads included in the core fungal community at the Bray-Curtis cut-offs for ab) roots and cd) soils of switchgrass monocultures of the Marginal Land Experiment. The red line represents the chosen threshold of a 5% increase in Bray-Curtis.


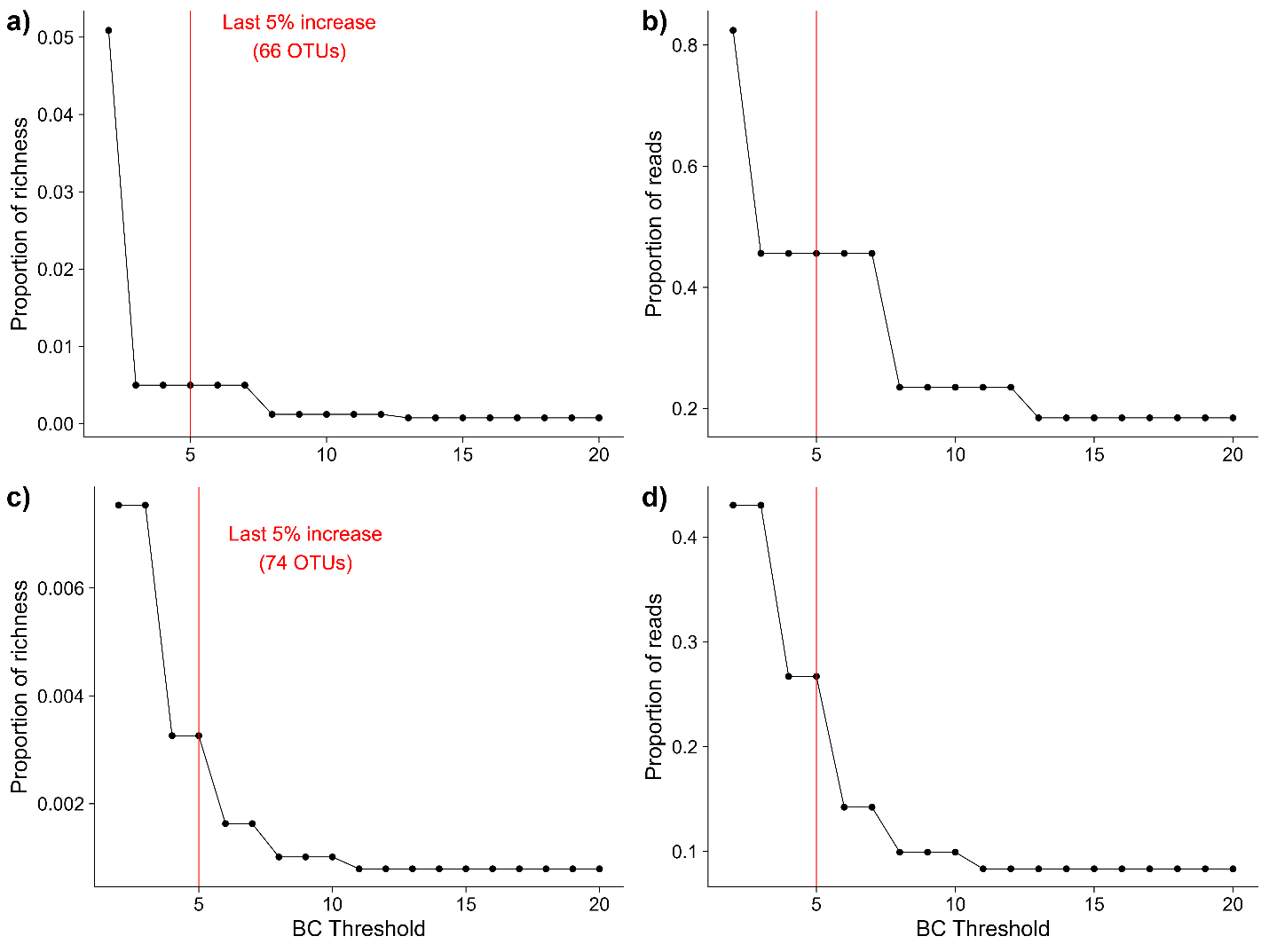


Figure S7. The proportion of ac) richness and bd) reads included in the core bacterial community at the Bray-Curtis cut-offs for ab) roots and cd) soils of switchgrass monocultures across one growing season at Lux Arbor. The red line represents the chosen threshold of a 5% increase in Bray-Curtis.


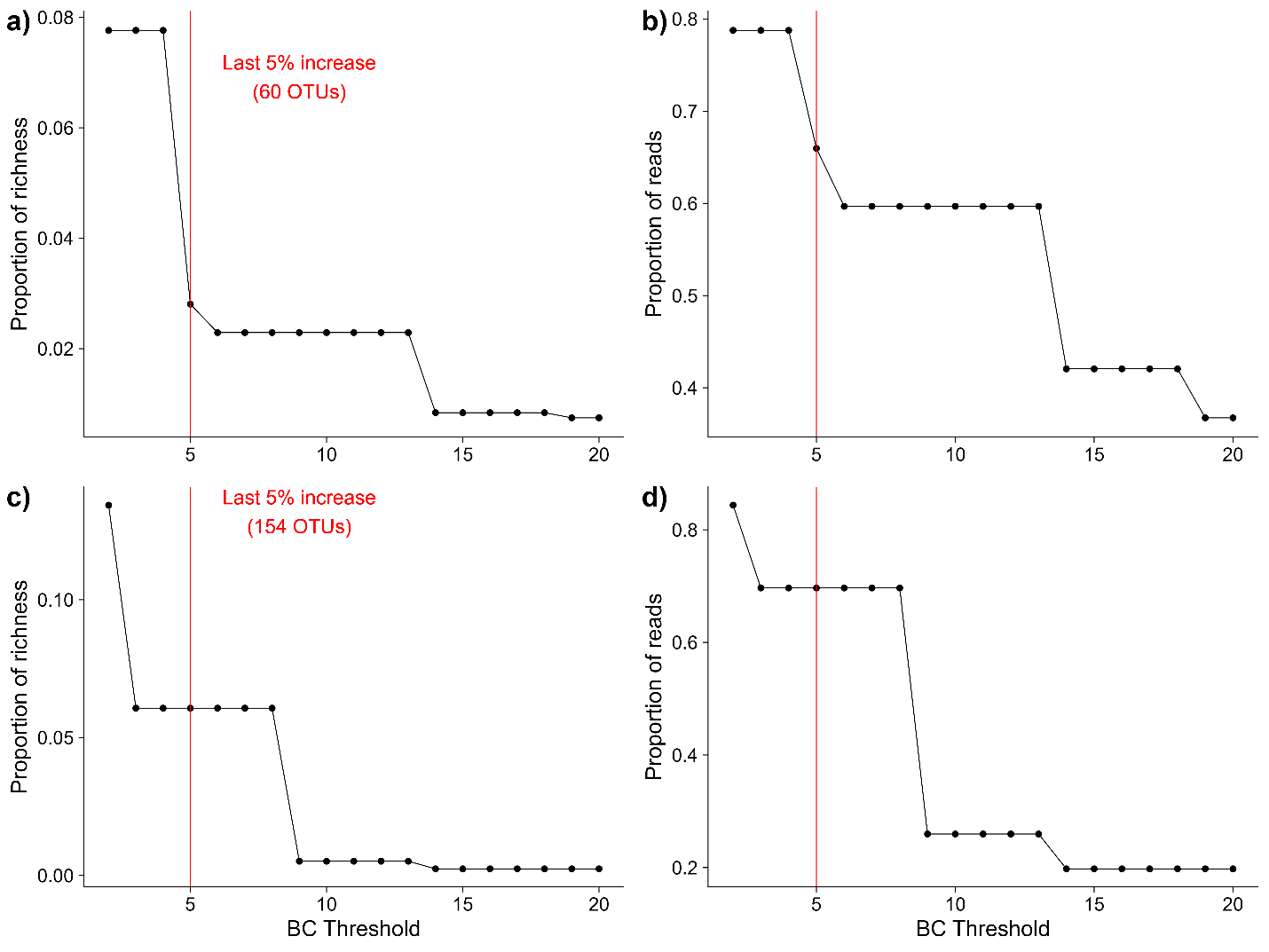


Figure S8. The proportion of ac) richness and bd) reads included in the core fungal community at the Bray-Curtis cut-offs for ab) roots and cd) soils of switchgrass monocultures across one growing season at Lux Arbor. The red line represents the chosen threshold of a 5% increase in Bray-Curtis.


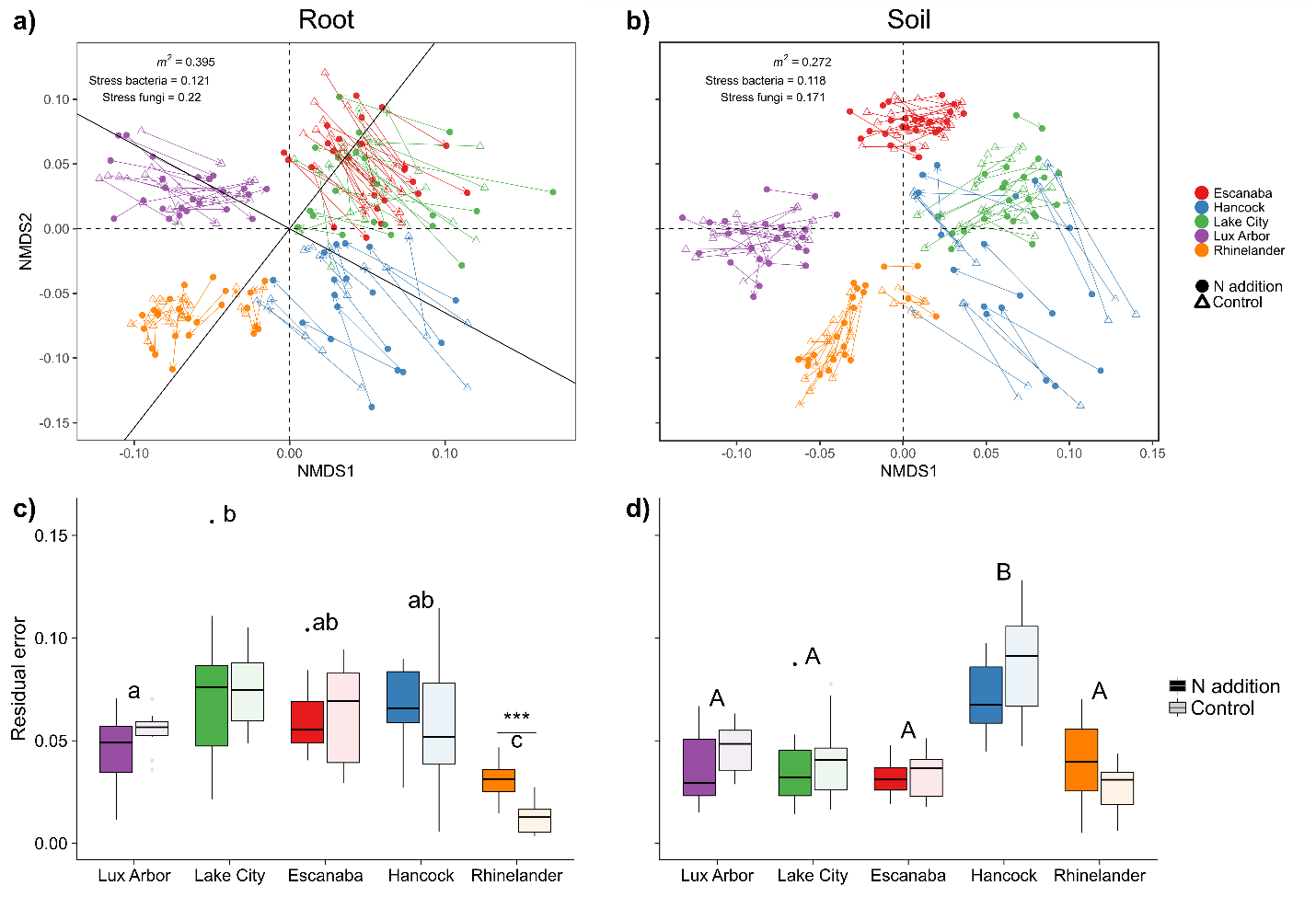


Figure S9. NMDS plots with Procrustes rotations of the fungal communities onto bacterial communities of a) root and b) soils of switchgrass monocultures at the Marginal Land Experiment in nitrogen (N) addition (filled circles) and control (open triangles) subplots sampled July 2018. Boxplot of the residual errors from the Procrustes models for c) roots and d) soils in N addition (dark fill) and control (light fill) subplots. Lowercase letters represent significant differences between sites for roots. Uppercase letters represent significant differences between sites for soil. ”***” represents *p* < 0.001 Tukey HSD adjusted significance for the effect of N addition.


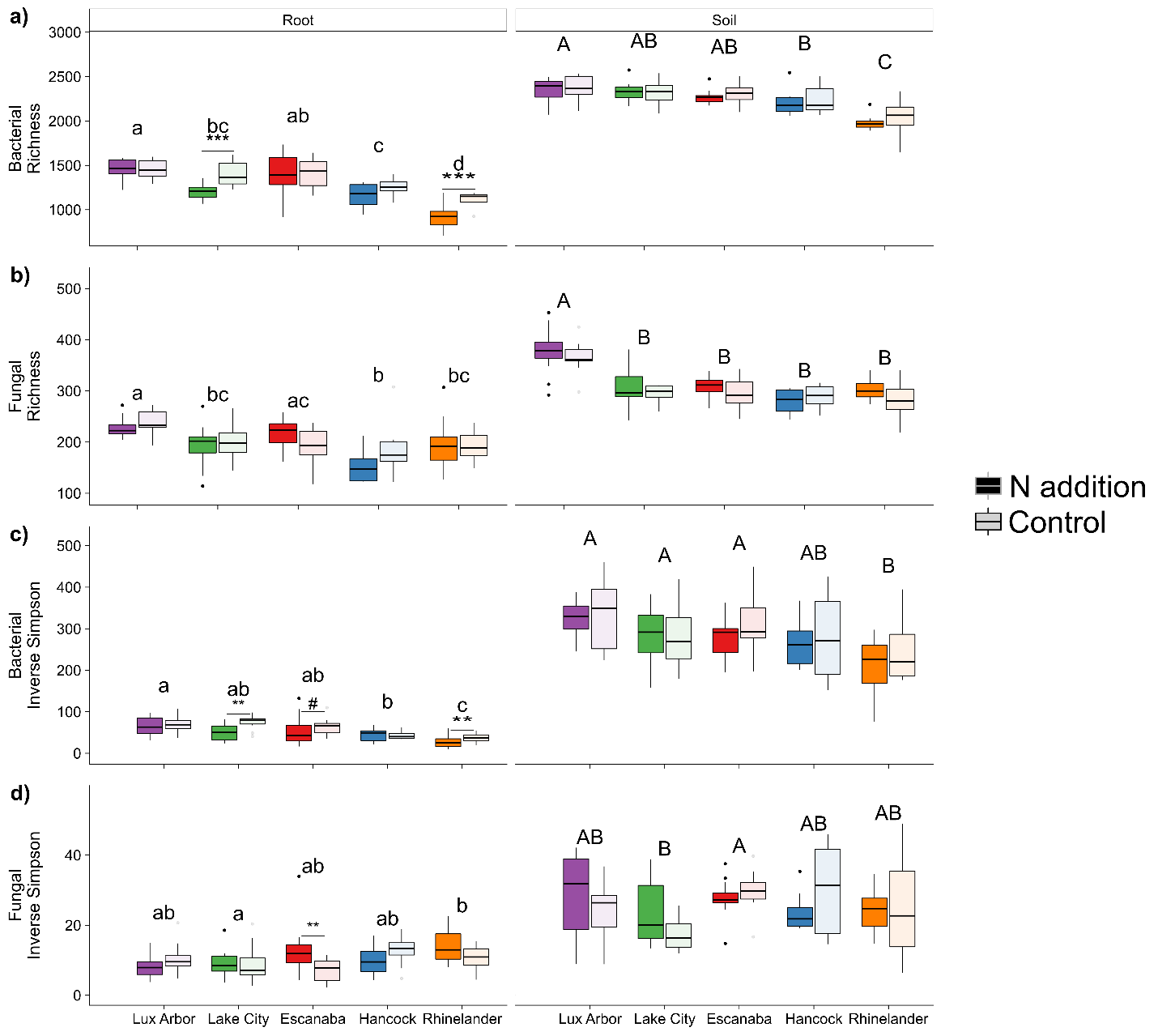


Figure S10. a) Bacterial richness, b) fungal richness, c) bacterial diversity, and d) fungal diversity (inverse Simpson) of switchgrass monocultures at each Marginal Land Experiment site from nitrogen (N) addition (dark fill) and control (light fill) subplots. Lowercase letters represent significant differences between sites for roots. Uppercase letters represent significant differences between sites for soil. “#”, ”**”, and ”***” represents *p* < 0.10, *p* < 0.01, and *p* < 0.001 Tukey HSD adjusted significance for the effect of N addition.


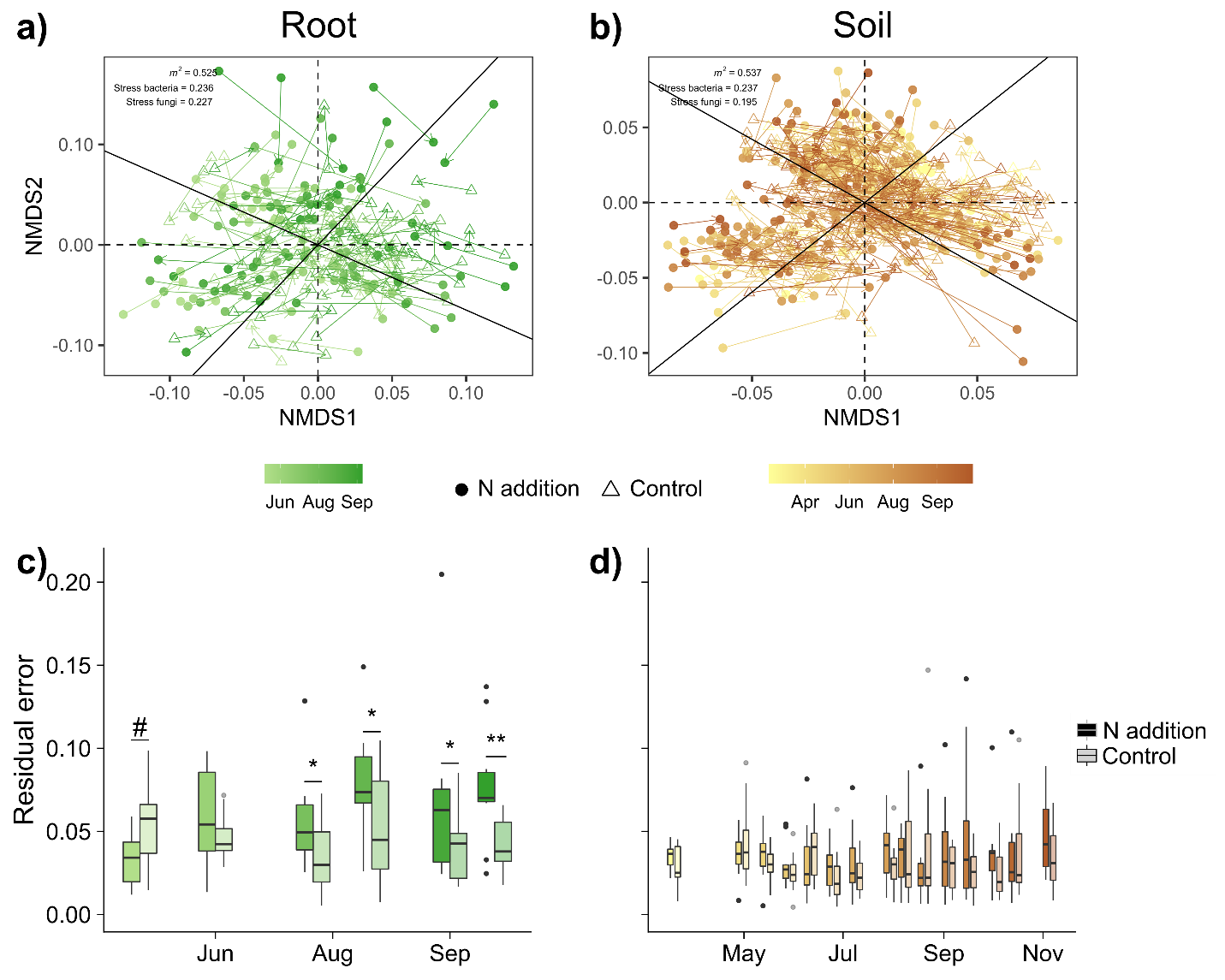


Figure S11. NMDS plots with Procrustes rotations of the fungal communities onto bacterial communities of a) root and b) soils of switchgrass monocultures at Lux Arbor from across one growing season in nitrogen (N) addition (filled circles) and control (open triangles) subplots. Boxplot of the residual errors from the Procrustes models for c) roots and d) soils from N addition (dark fill) and control (light fill) subplots. Fill color ramp represents collection dates with lighter colors representing earlier dates and darker colors later dates. “#”, ”*”, and ”**” represents *p* < 0.10, *p* < 0.05, and *p* < 0.01 Tukey HSD adjusted significance for the effect of N addition.
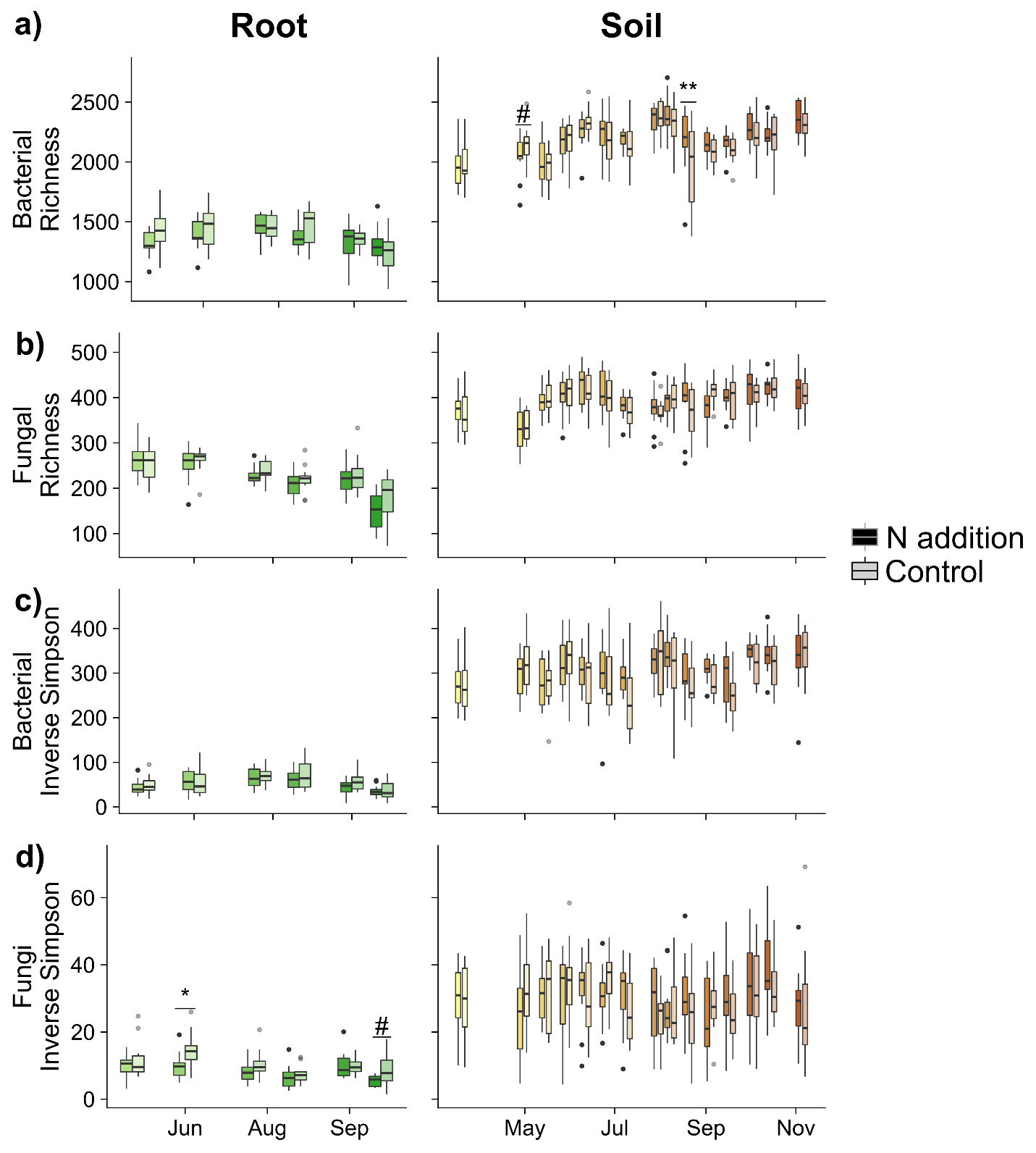
Figure S12. a) Bacterial richness, b) fungal richness, c) bacterial diversity, and d) fungal diversity (inverse Simpson) of switchgrass monocultures across one growing season at Lux Arbor in nitrogen (N) addition (dark fill) and control (light fill) subplots. Fill color ramp represents collection dates with lighter colors representing earlier dates and darker colors representing later dates. “#”, ”*”, and ”**” represents *p* < 0.10, *p* < 0.05, and *p* < 0.01 Tukey HSD adjusted significance for the effect of N addition.


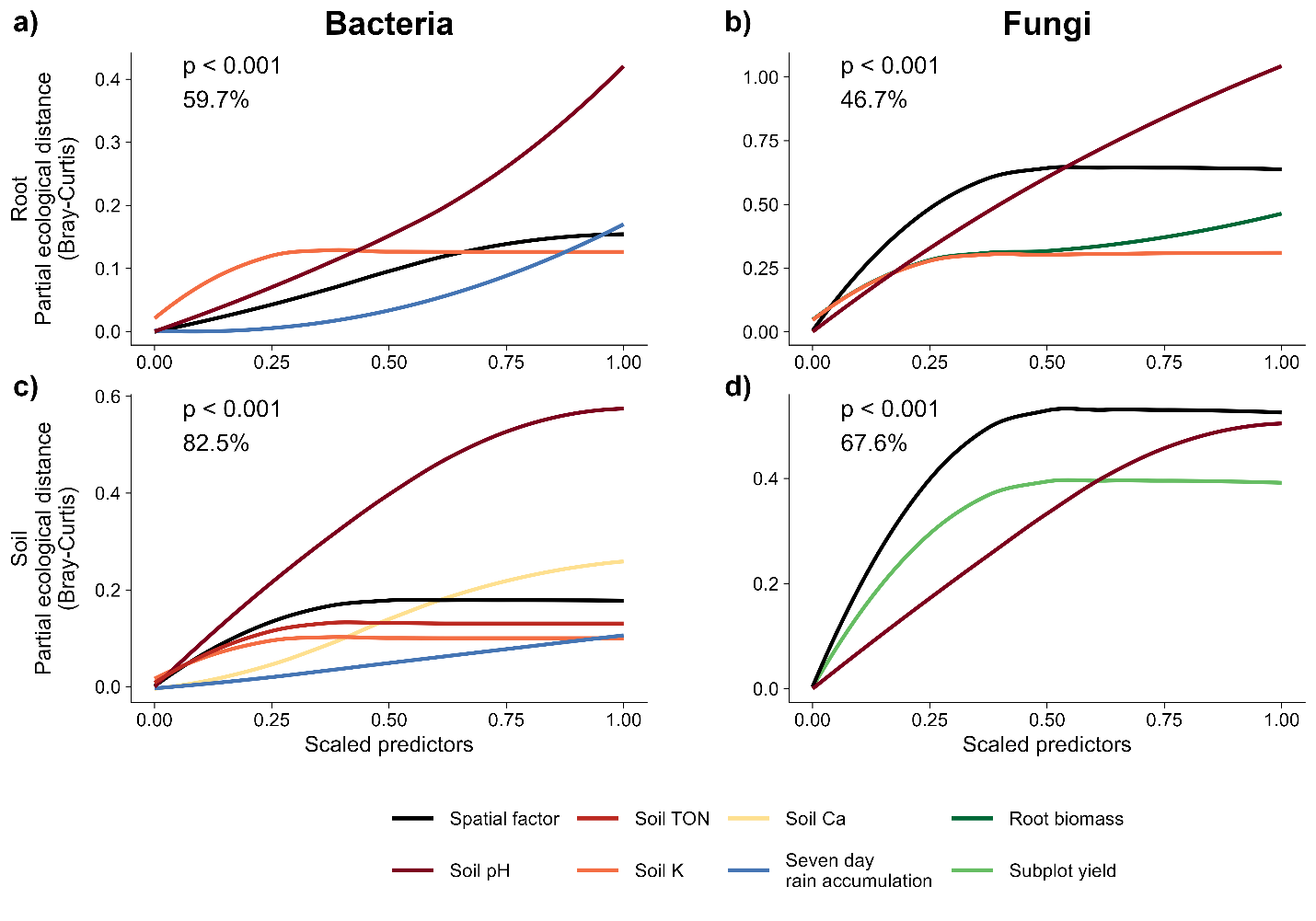


Figure S13. Loess graphs from generalized dissimilarity models (GDMs) of the a) root bacterial, b) root fungal, c) soil bacterial, and d) soil fungal communities of switchgrass monocultures from sites of the Marginal Land Experiment. Variables were scaled (0 to 1) to allow for comparison. Panel text represents the significance and percentage of deviance explained by GDMs.


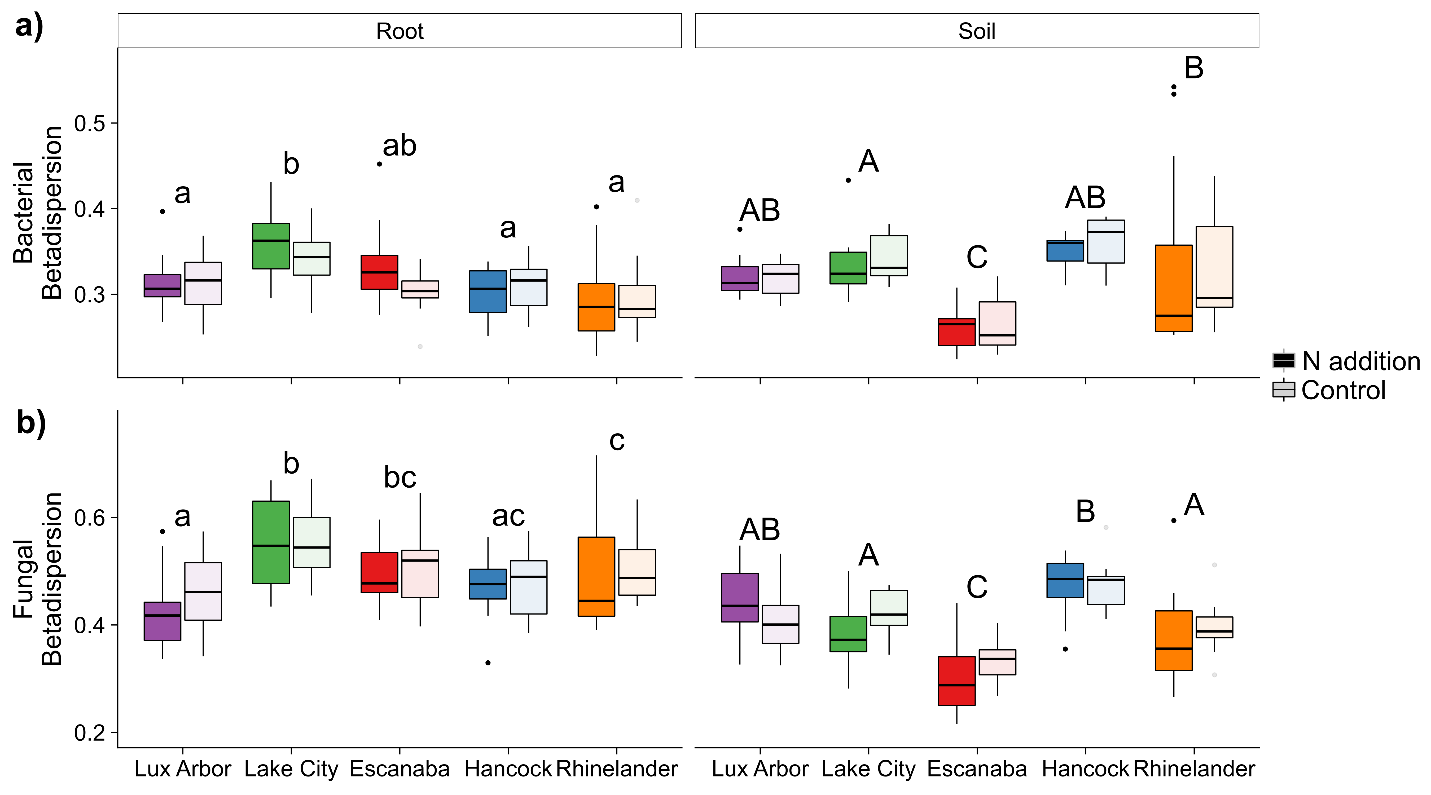


Figure S14. Beta dispersion in a) bacterial and b) fungal communities of the roots and soils of switchgrass monocultures at the Marginal Land Experiment sites from nitrogen (N) addition (dark fill) and control (light fill) subplots. Lowercase letters represent significant differences between sites for roots. Uppercase letters represent significant differences between sites for soil. Nitrogen addition did not have a significant effect on beta dispersion.


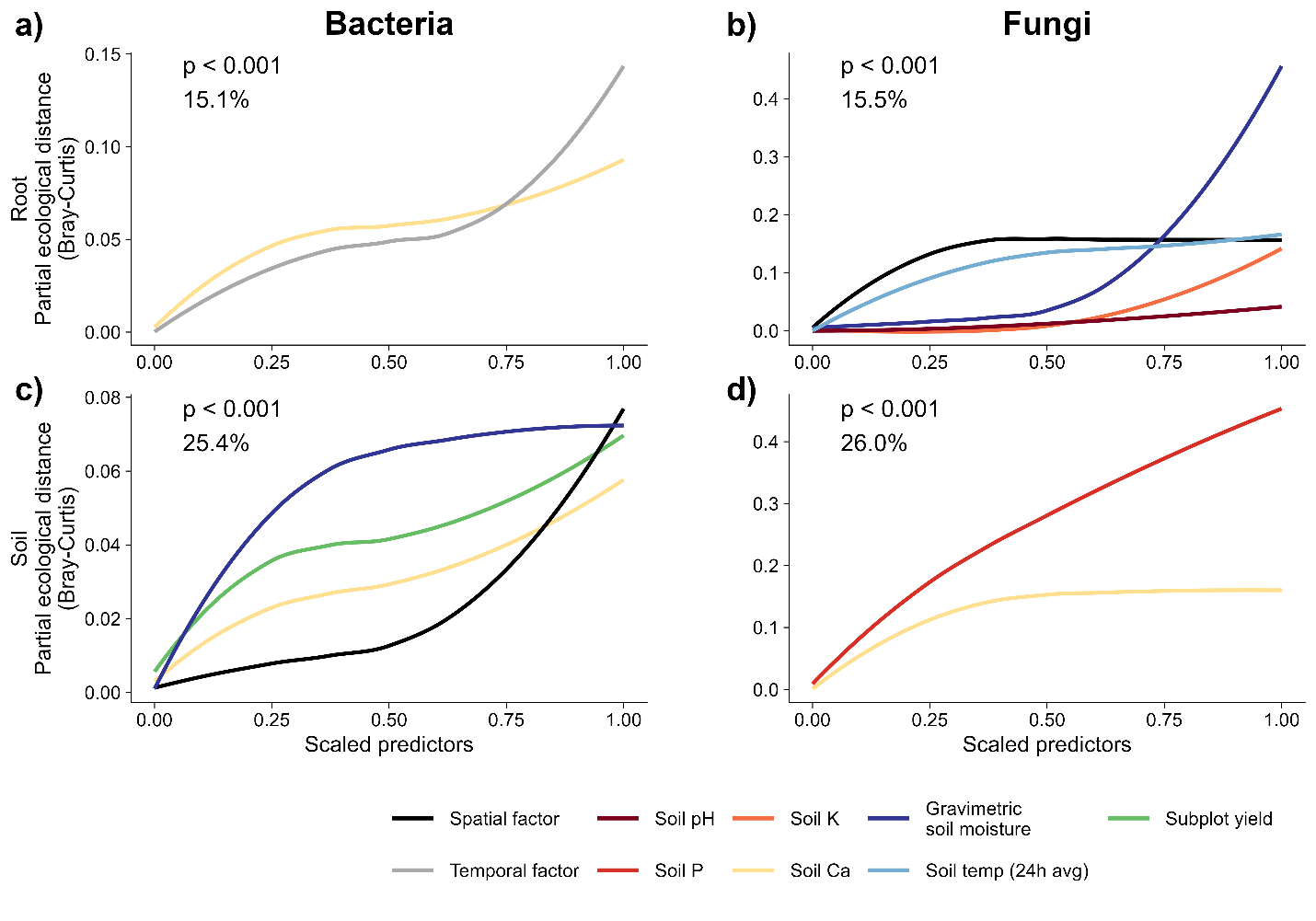


Figure S15. Loess graphs from generalized dissimilarity models (GDMs) of the a) root bacterial, b) root fungal, c) soil bacterial, and d) soil fungal communities of switchgrass monocultures from Lux Arbor across one growing season. Variables were scaled (0 to 1) to allow for comparison. Panel text represents the significance and percentage of deviance explained by GDMs.


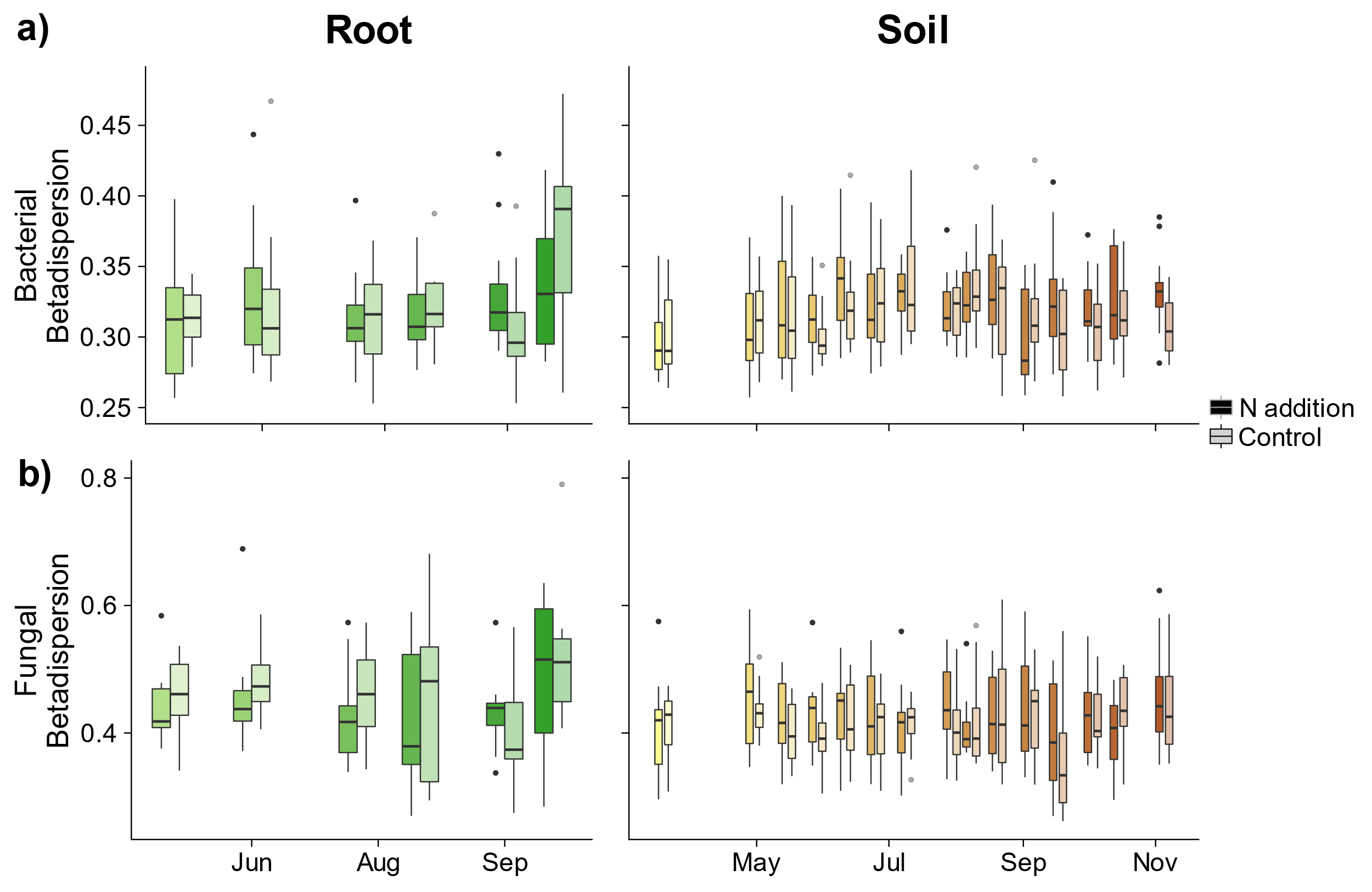


Figure S16. Beta dispersion in a) bacterial and b) fungal communities of the roots and soils of switchgrass monocultures at Lux Arbor from N addition (dark fill) and control (light fill) subplots across one growing season. Fill color ramp represents collection dates with lighter colors representing earlier dates and darker colors later dates. Nitrogen addition did not have a significant effect on beta dispersion.


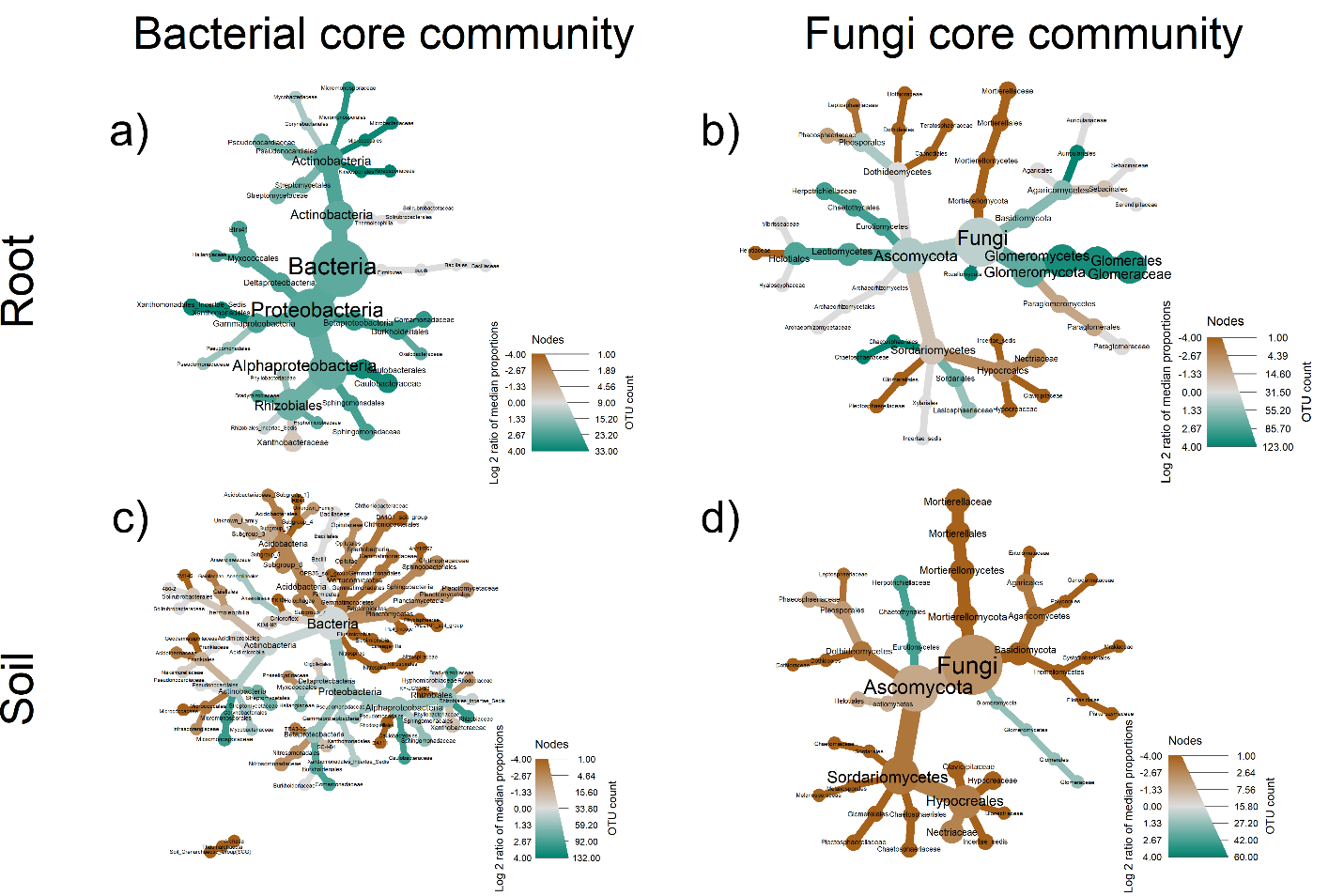


Figure S17. Heat phylogenetic trees of families within core a) root bacterial, b) root fungal, c) soil bacterial, and d) soil fungal communities of switchgrass monocultures from sites of the Marginal Land Experiment. Node size represents richness (# of OTUs) and node color represents the log2 ratio of median proportions of read abundance between root (green colors) or soil (brown colors) communities. Grey nodes represent non-significant differences in taxa abundance (*p* > 0.05; false discovery rate adjusted Wilcoxon rank-sum pairwise test).


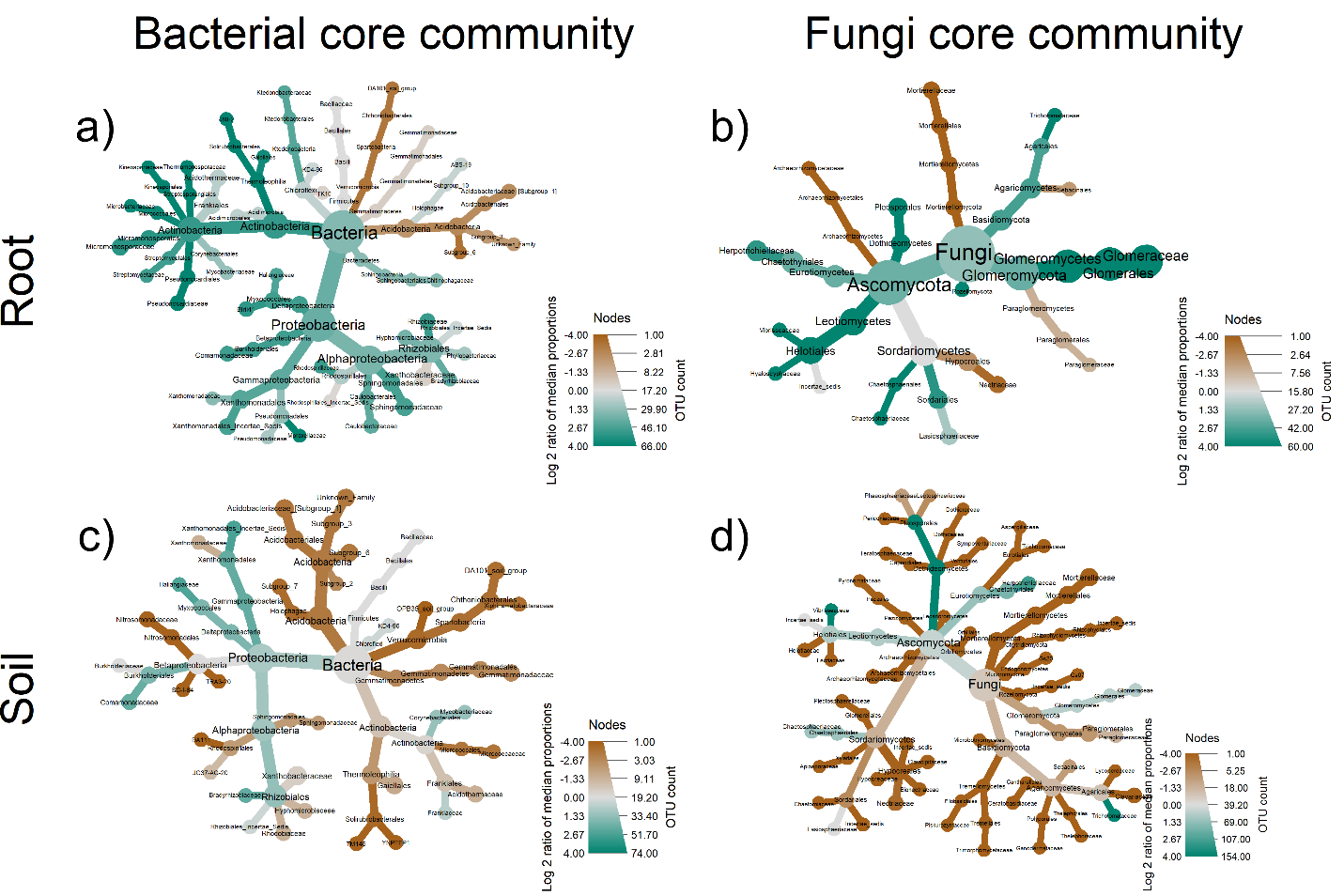
Figure S18. Heat phylogenetic tree of families within core a) root bacterial, b) root fungal, c) soil bacterial, and d) soil fungal communities of switchgrass monocultures from Lux Arbor across one growing season. Node size represents richness (# of OTUs) and node color represents the log2 ratio of median proportions of read abundance between root (green colors) or soil (brown colors) communities. Grey nodes represent non-significant differences in taxa abundance (*p* > 0.05; false discovery rate adjusted Wilcoxon rank-sum pairwise test).


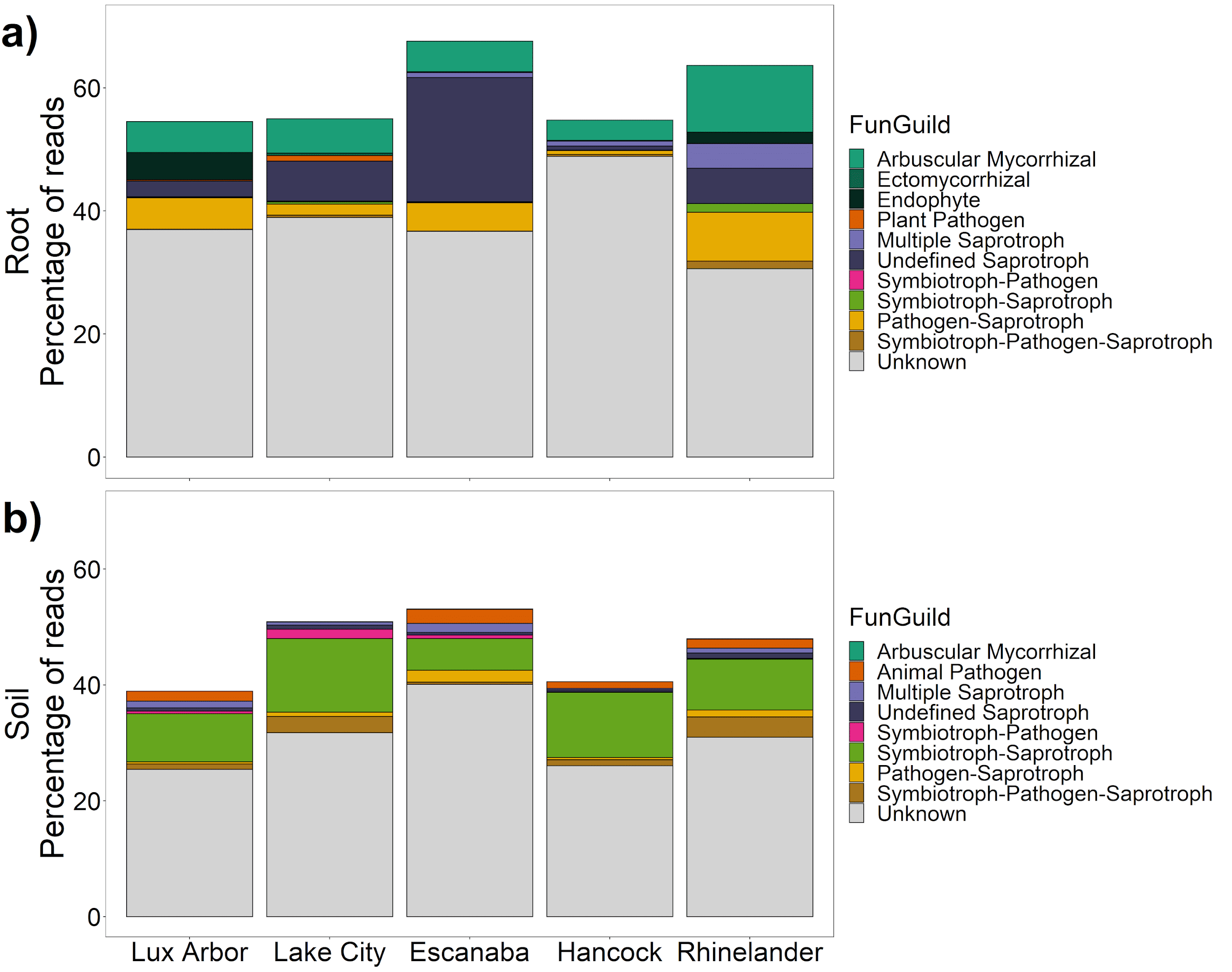


Figure S19. Stacked bar graphs of the FunGuild classifications of core a) root and b) soil fungal communities of switchgrass monocultures at sites of the Marginal Land Experiment. Guilds with multiple hits are grouped.


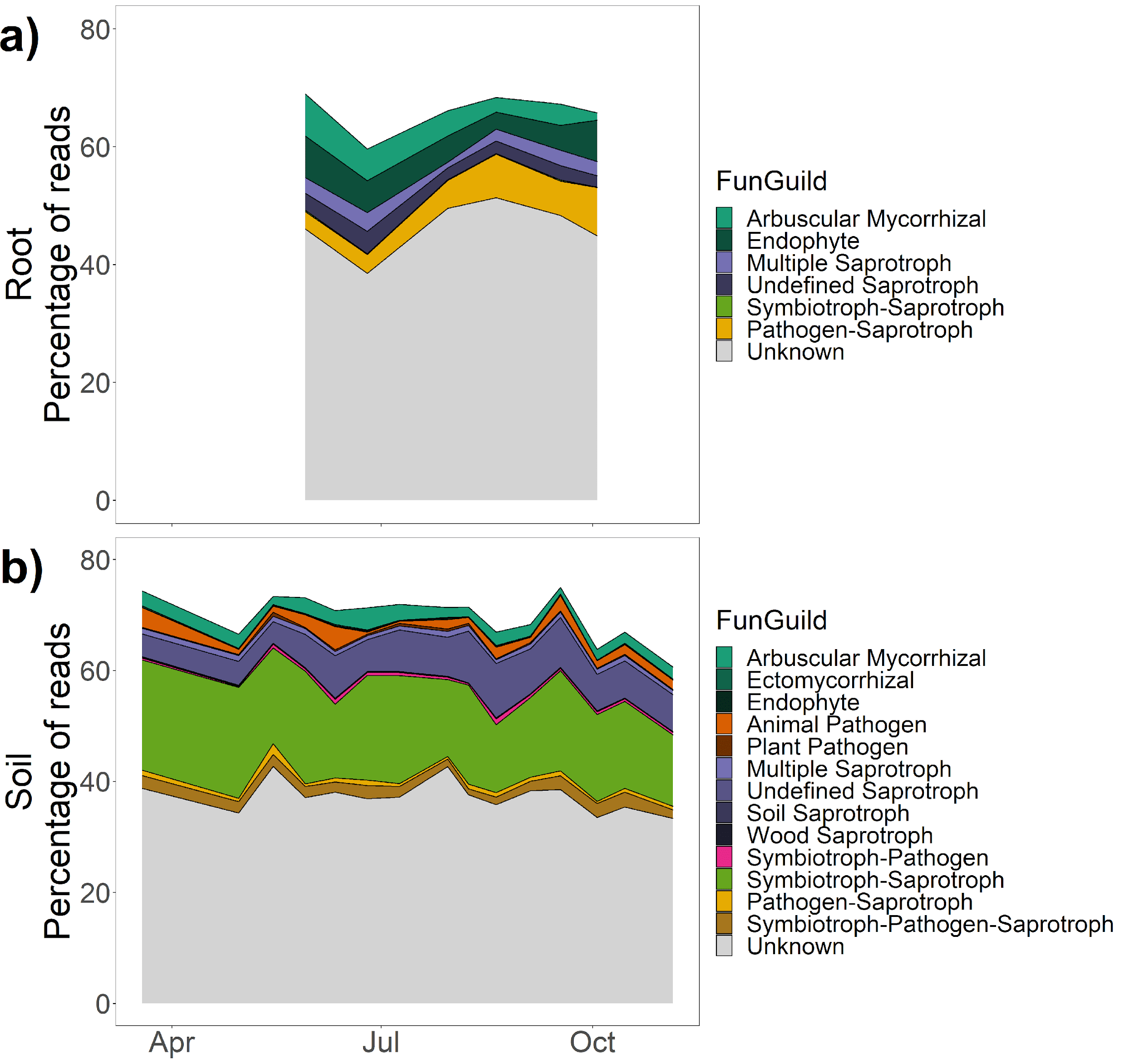


Figure S20. Stacked bar graphs of the FunGuild classifications of core a) root and b) soil fungal communities of switchgrass monocultures from Lux Arbor across one growing season. Guilds with multiple hits are grouped.
